# Fatty Acid Binding Protein 3 And Transzonal Projections Are Involved In Lipid Accumulation During *In Vitro* Maturation Of Bovine Oocytes

**DOI:** 10.1038/s41598-017-02467-9

**Published:** 2017-06-01

**Authors:** Maite del Collado, Juliano Coelho da Silveira, Juliano Rodrigues Sangalli, Gabriella Mamede Andrade, Letícia Rabello da Silva Sousa, Luciano Andrade Silva, Flavio Vieira Meirelles, Felipe Perecin

**Affiliations:** Veterinary Medicine Department, Faculty of Animal Sciences and Food Engineering, University of Sao Paulo, Av. Duque de Caxias Norte 225, 13635-900 Pirassununga, SP Brazil

## Abstract

Oocytes that undergo *in vitro* maturation (IVM) are metabolically abnormal and accumulate excess lipid content. However, the mechanism of lipid accumulation and the role of cumulus cells in this process are unclear. Recently, it was shown that fatty acid binding proteins (FABPs) performed intra- and extracellular fatty acid transport. We postulated that FABP3 might be responsible for fatty acid transport from cumulus cells to the oocytes *via* transzonal projections (TZPs) during IVM. Transcript and protein levels of FABP3 were analyzed in both *in vivo*- and *in vitro*-matured cumulus-oocyte-complexes and were increased in IVM samples. Further analysis showed increased lipid content in oocytes and cumulus cells in IVM samples compared to *in vivo*-derived. We therefore speculated that altered traffic of fatty acids *via* FABP3 during IVM was the mechanism leading to the excess of lipids accumulated within IVM oocytes. Furthermore, we demonstrated an increase in FABP3 levels and lipid content during the first 9 h of IVM, further strengthening the possibility of fatty acid transport *via* FABP3 and TZPs. Additionally, disruptions of TZPs during IVM decreased lipid accumulation in oocytes. Our results shed light on a possible mechanism involving FABP3 and TZPs that causes excess lipid accumulation in oocytes during IVM.

## Introduction

Assisted reproductive technologies (ARTs) expose gametes and embryos to non-physiological conditions that may cause abnormal development. Increased lipid accumulation during *in vitro* production (IVP) of bovine embryos is one of the most well recognized metabolic abnormalities that accompany the outcome of successful ARTs. The negative effects of increased lipid content in blastomeres include reduced potential for cryopreservation and poor post-thawing embryo survival rates, resulting in low rates of pregnancy and embryo losses^1, 2^. Studies on bovine embryos produced using different IVP systems have provided compelling evidence for correlation between lipid-rich culture media supplemented with fetal bovine serum (FBS) and high lipid content as well as low cryotolerance of blastocysts^3–5^. It was demonstrated that the lipid accumulation in species such as bovine and mouse occurs during the first step of IVP, the *in vitro* maturation (IVM) of oocytes^3, 6^, and that it does not occur when the maturation takes place *in vivo* ^3^. Furthermore, the lipid accumulation was accentuated when the oocytes were matured in the presence of FBS^3^. Additionally, similar events were observed *in vivo*, when cumulus-oocyte complexes (COCs) were exposed to lipid-rich environments such as follicular fluids of obese women; this exposure may be responsible for excessive lipid accumulation in oocytes, leading to impaired fertility^7–11^.

Communication between cumulus cells and oocytes is important for optimal oocyte maturation. This communication is mediated by paracrine signals, transzonal projections (TZPs), gap junctions and possibly extracellular vesicles^12, 13^. Despite the paracrine signals, molecules could be transported within TZPs and be transferred to the oocyte by GAP junctions or by the recently proposed extracellular vesicle mediated intercellular transport^13, 14^. Gap junctions can transport low-weight molecules (<1 kDa) important for oocyte development, such as ions, nucleotides, amino acids, and metabolites, and are functional during the first 6 h of IVM^15, 16^. TZPs are large channels, ~2 μm in diameter, that allow the transport of large molecules such as mRNAs to oocytes until ~9 h after the resumption of meiosis^13^. Exchange of lipids between cumulus cells and oocytes or lipid carriers for fatty acid transport have not been observed in this system so far.

Fatty acid molecules are transported intra- and extracellularly by a family of lipid-binding proteins called Fatty Acid Binding Proteins (FABP); 9 isoforms of FABPs have been reported^17^. These proteins bind mostly to long chain fatty acids (C16-C20) and transport them to the peroxisome, mitochondria or endoplasmic reticulum^18–20^. FABPs also transport lipids extracellularly, and have been observed free or enclosed in extracellular vesicles in several tissues and body fluids; they thus serve pleiotropic functions in systemic metabolism^17, 21^. Isoform 3 of these lipid-binding proteins, called FABP3 or heart-FABP, seems to have altered functionality within COCs because its transcript levels were reported to be increased during IVM^22^.

Even though the dynamic interaction between cumulus cells and oocytes as well as the transport of several molecules into COCs are well known, there is still a gap in the knowledge of the mechanism of lipid metabolism in these complexes during *in vivo* or IVM. In this study, we used an animal model to investigate lipid transport in COCs during oocyte maturation. This model allowed us to study the roles of FABP3 and TZPs in lipid accumulation as well as the influence of the *in vitro* environment imposed by ARTs on the lipid accumulation in COCs.

We demonstrated the transport of FABP3 between cumulus cells and oocytes through TZPs during IVM. This traffic is maintained until around 9 h after the beginning of IVM, and is consistent with the period at which higher FABP3 expression and lipid accumulation was observed in the oocytes. Additionally, blocking TZP formation resulted in reduced lipid droplets content in oocytes undergoing IVM. Our data also suggested that IVM altered the FABP3-mediated accumulation of lipids in COCs. To the best of our knowledge, this is the first manuscript to describe a mechanism of lipid transport within COCs. The data presented here will provide new insights that help elucidate the mechanism behind lipid accumulation during IVM and potentially lead to the development of new approaches to improve ARTs.

## Results

### IVM leads to increased lipid content in COCs

**Figure 1:**
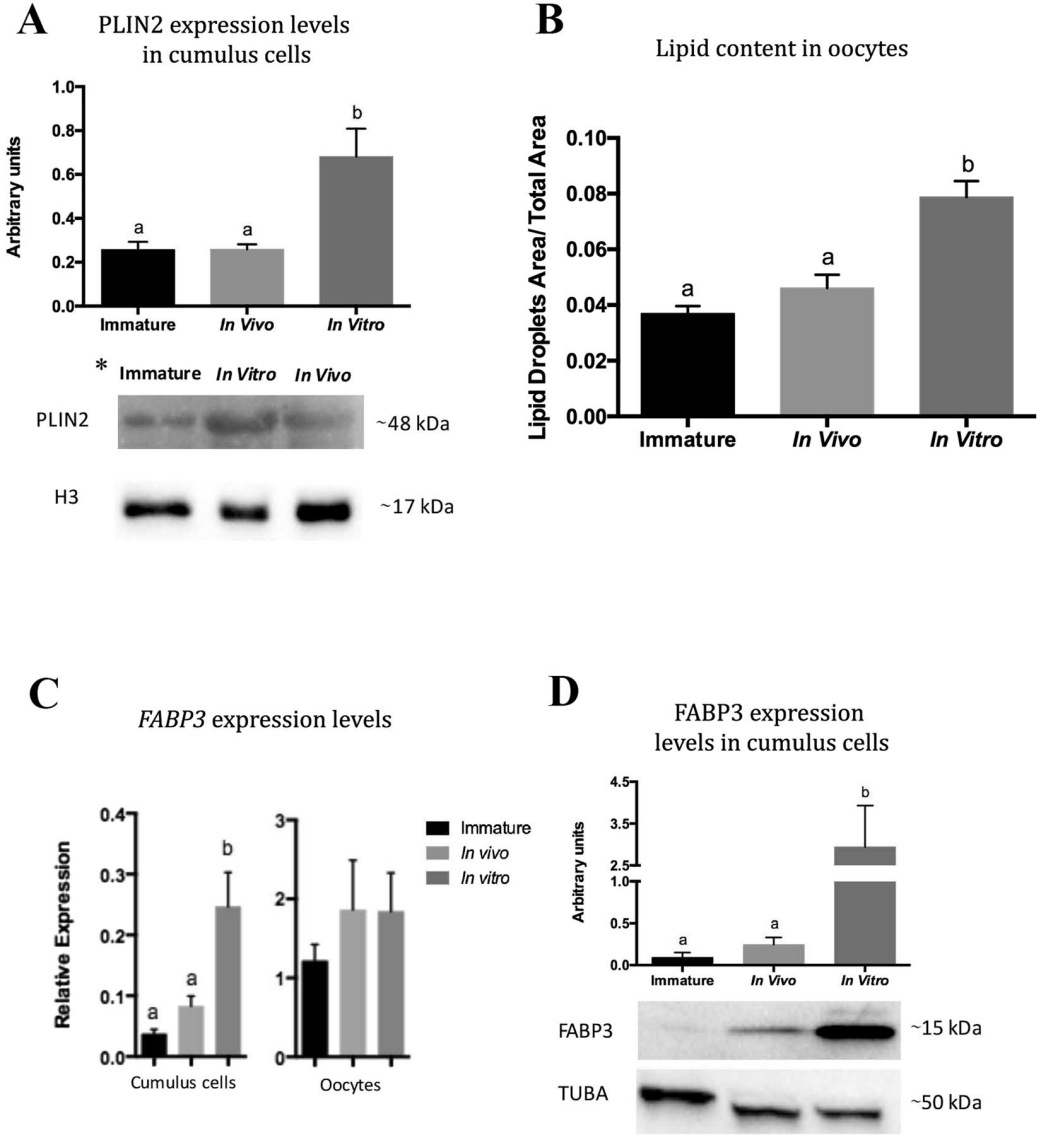
Quantification of lipid content in cumulus cells and oocytes derived from immature, *in vivo*-matured and *in vitro*-matured cumulus-oocyte complexes (COCs). (**A**) Lipid content in cumulus cells was determined by western blot analysis of perilipin 2 (PLIN2) using histone 3 (H3) as a normalizer. (**B**) Lipid quantification in oocytes was performed by fluorescence confocal microscopy of lipid droplets. The values for lipid content represent the ratio of area of lipid droplets to total oocyte area. Representative immature, *in vivo*-matured and *in vitro*-matured oocytes stained with BODIPY 493/503 are shown in Supplementary Fig. S1. (**C**) Relative amounts of fatty acid binding protein 3 (*FABP3)* transcripts in immature, *in vivo*-, and *in vitro*- matured cumulus cells and oocytes. (**D**) FABP3 protein levels in cumulus cells normalized by x-Tubulin (TUBA). Lowercase letters above bars in the same graph indicate significant differences (*P* *<* 0.05). Values are presented as mean ± standard error of the mean. The full western blot images in (**A**) and (**D**) are shown in Supplementary Fig. S2a and S2b, respectively. *The order of the groups is different between the graph and the representative immunoblot images.

To investigate the dynamics of lipid accumulation *in vivo* and IVM, we estimated the lipid content in cumulus cells and oocytes. Higher amounts of lipid droplets were observed in both cumulus cells (Fig. 1a and Supplementary Fig. S2a) and oocytes obtained after IVM (Fig. 1b and Supplementary Fig. S1). The results demonstrated that IVM caused an increase in lipid content, whereas *in vivo* cumulus cells and oocytes had lipid content similar to that in the immature group.

### IVM increases FABP3 transcript and protein levels in cumulus cells

In order to investigate the effect of IVM on FABP3 function, we determined the transcript and protein levels of FABP3 in cumulus cells derived from immature, *in vivo*- and *in vitro*-matured COCs. We observed that the relative amount of FABP3 transcripts increased in cumulus cells after IVM of COCs (Fig. 1c). Interestingly, no difference was observed in the relative levels of FABP3 transcripts in oocytes before and after IVM (Fig. 1c). Similarly, FABP3 protein levels in cumulus cells obtained from COCs that underwent IVM were also found to be higher than those in immature and *in vivo*-matured cells (Fig. 1d and Supplementary Fig. S2b). Our data demonstrated that *in vitro*-matured COCs had higher transcript and protein levels of FABP3 in cumulus cells.

### FABP3 is localized within TZPs during IVM

**Figure 2:**
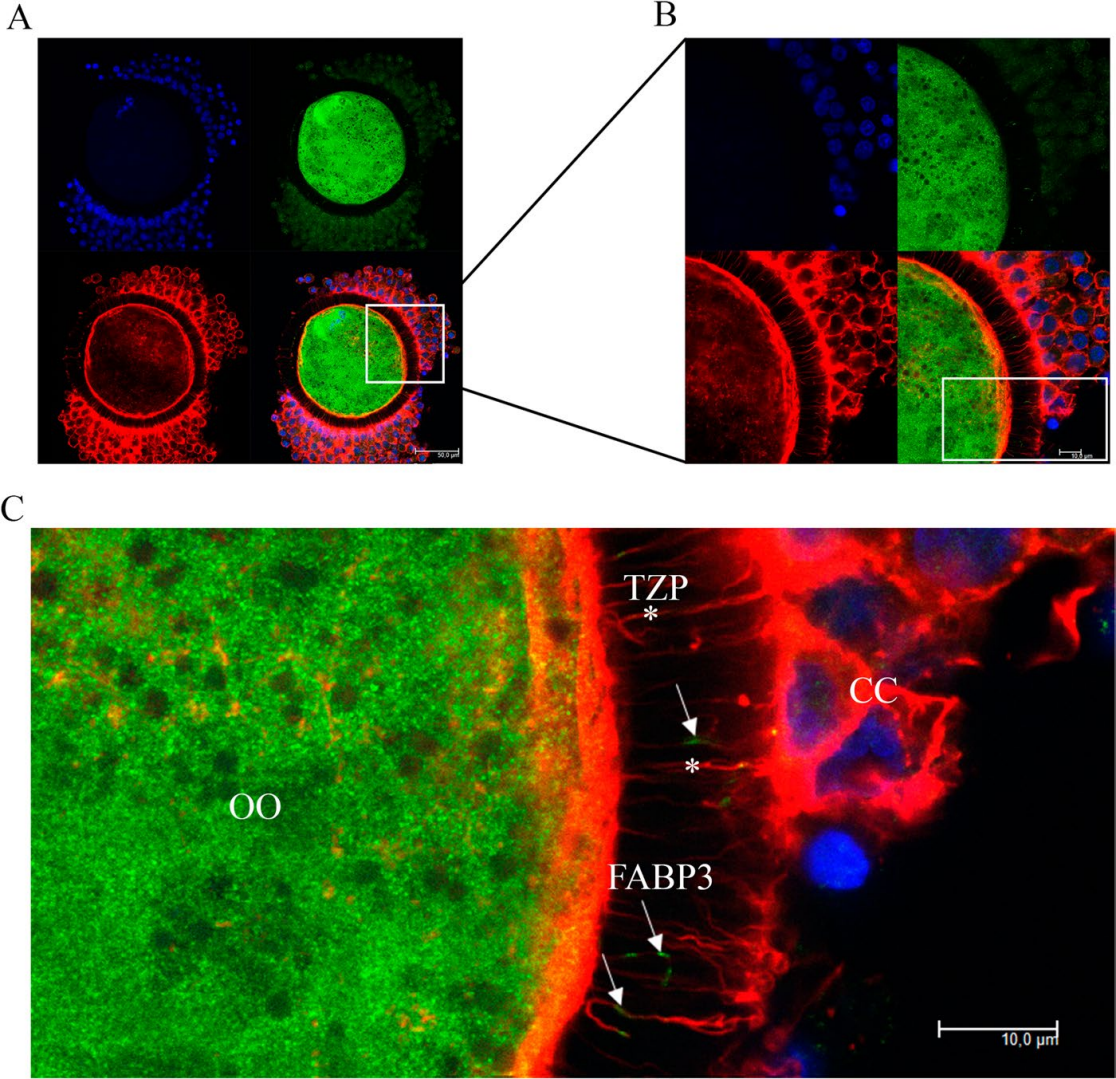
Confocal microscopic analysis demonstrating the presence of fatty acid binding protein 3 (FABP3) within the transzonal projections (TZPs) of immature cumulus-oocyte-complexes (COCs). FABP3 was immunostained using 488 Alexa Fluor (green); TZPs (actin) were labeled with Alexa Fluor 647 phalloidin (red) and nuclei were stained with DAPI (blue). (**A**) Photomicrographs of immature COCs were obtained in 63 x objective and (**B**) in 63 x objective zoomed 3.5 x. (**C**) Digital zoom showing FABP3 (arrow) within TZPs (asterisk) in zona pellucida (OO = oocyte; CC = cumulus cells). A total of 12 immature COCs were analysed and showed similar pattern; 6 additional COCs are shown in Supplementary Fig. S3.

**Figure 3:**
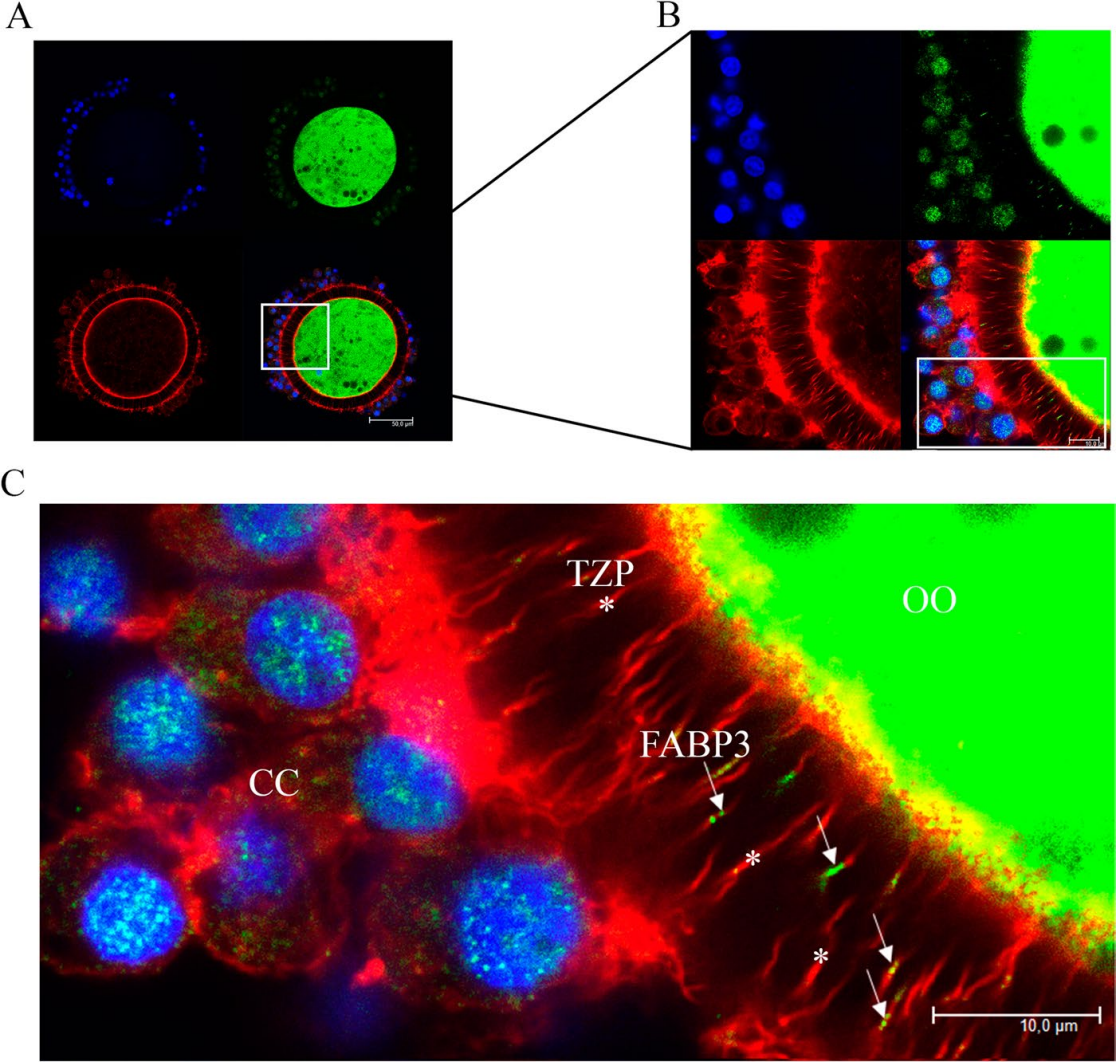
Confocal photomicrographs of fatty acid binding protein 3 (FABP3) immunodetection in transzonal projections (TZPs) after 9 h of *in vitro* maturation (IVM). FABP3 is immunostained using 488 Alexa Fluor (green); TZPs (actin) are stained with Alexa Fluor 647 phalloidin (red); nuclei are stained with DAPI (blue). (**A**) Photomicrographs of COCs after 9 h of IVM were obtained in 63x objective and (**B**) in 63x objective zoomed 3.5x. (**C**) Digital zoom showing FABP3 (arrow) along TZPs (asterisk) in the zona pellucida (OO = oocyte; CC = cumulus cells). A total of 12 COCs *in vitro*-matured for 9 hours were analysed and showed similar pattern; 6 additional COCs are shown in Supplementary Fig. S4.

**Figure 4:**
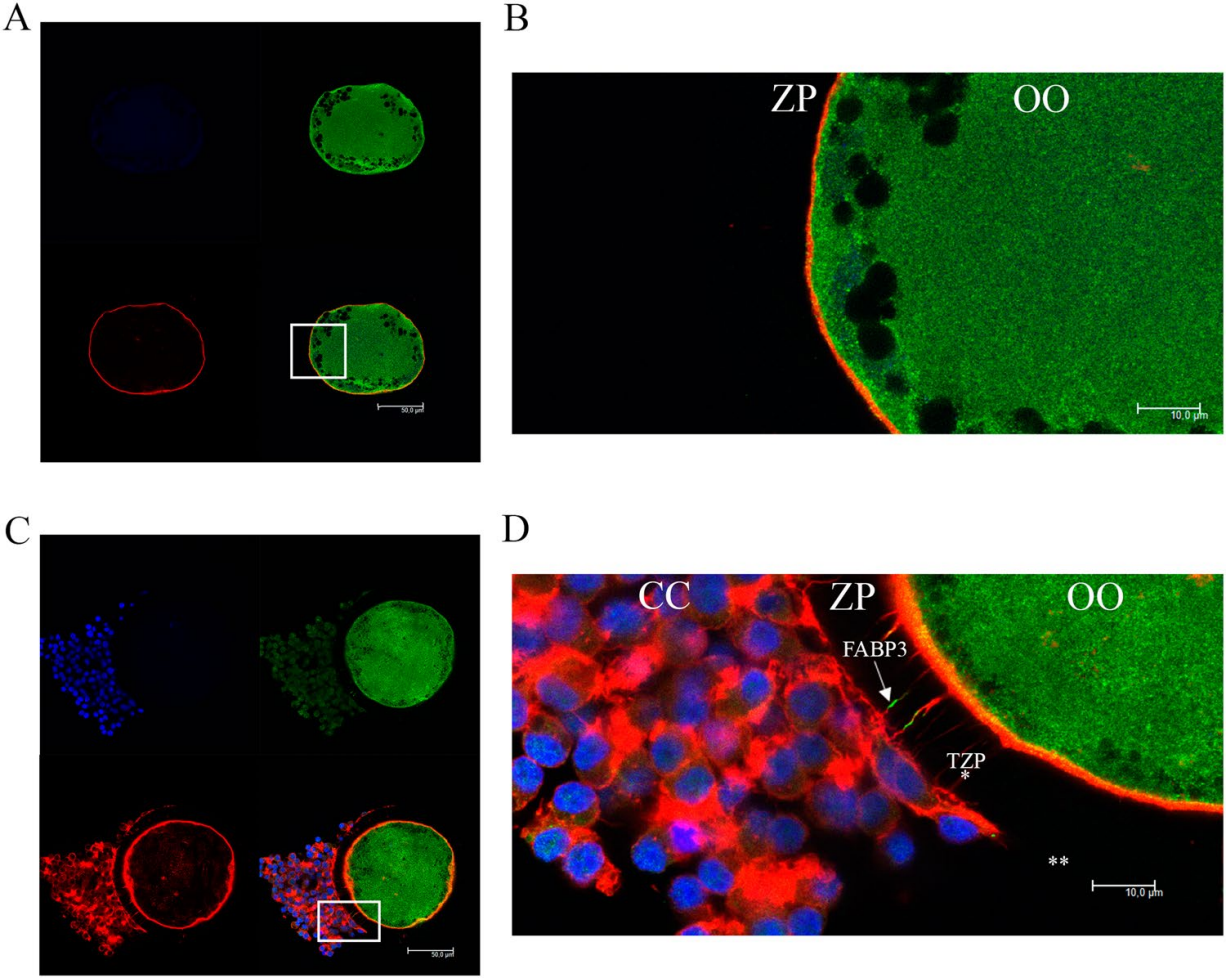
Confocal microscopic analysis of fatty acid binding protein 3 (FABP3) in tranzonal projections (TZPs) of denuded and partially denuded oocytes cultured in the maturation media for 9 h. (**A**) Images acquired from denuded oocytes show no FABP3 and TZPs within the zona pellucida. (**B**) The image of a portion of the denuded oocytes demonstrating the complete absence of TZPs and FABP3 within the zona pellucida. (**C**) Images acquired from a partially denuded oocyte demonstrating the presence of FABP3 at the same location as TZPs within the zona pellucida. (**D**) The portion of the partially denuded COCs demonstrating the complete absence of TZPs and FABP3 within the zona pellucida in the denuded area, and the presence of FABP3 and TZPs in the cumulus-enclosed area. FABP3 (arrow) is immunostained using 488 Alexa Fluor (green); TZPs (actin; asterisk) are stained with Alexa Fluor 647 phalloidin (red); nuclei are stained with DAPI (blue). Photomicrographs were obtained in 63x objective (**A** and **C**), and merged with z-stack captures with digital zoom (**B** and **D**) to illustrate the absence (**B**) or presence (**D**) of FABP3 along the TZPs. (OO = oocyte; CC = cumulus cells). ** indicates the lack of cumulus cells and TZPs. A total of 10 denuded oocytes and 11 partially denuded oocytes *in vitro*-matured for 9 hours were analysed and showed similar pattern; 6 additional denuded oocytes and 6 additional partially denuded oocytes are shown in Supplementary Figs S5 and S6, respectively.

**Figure 5:**
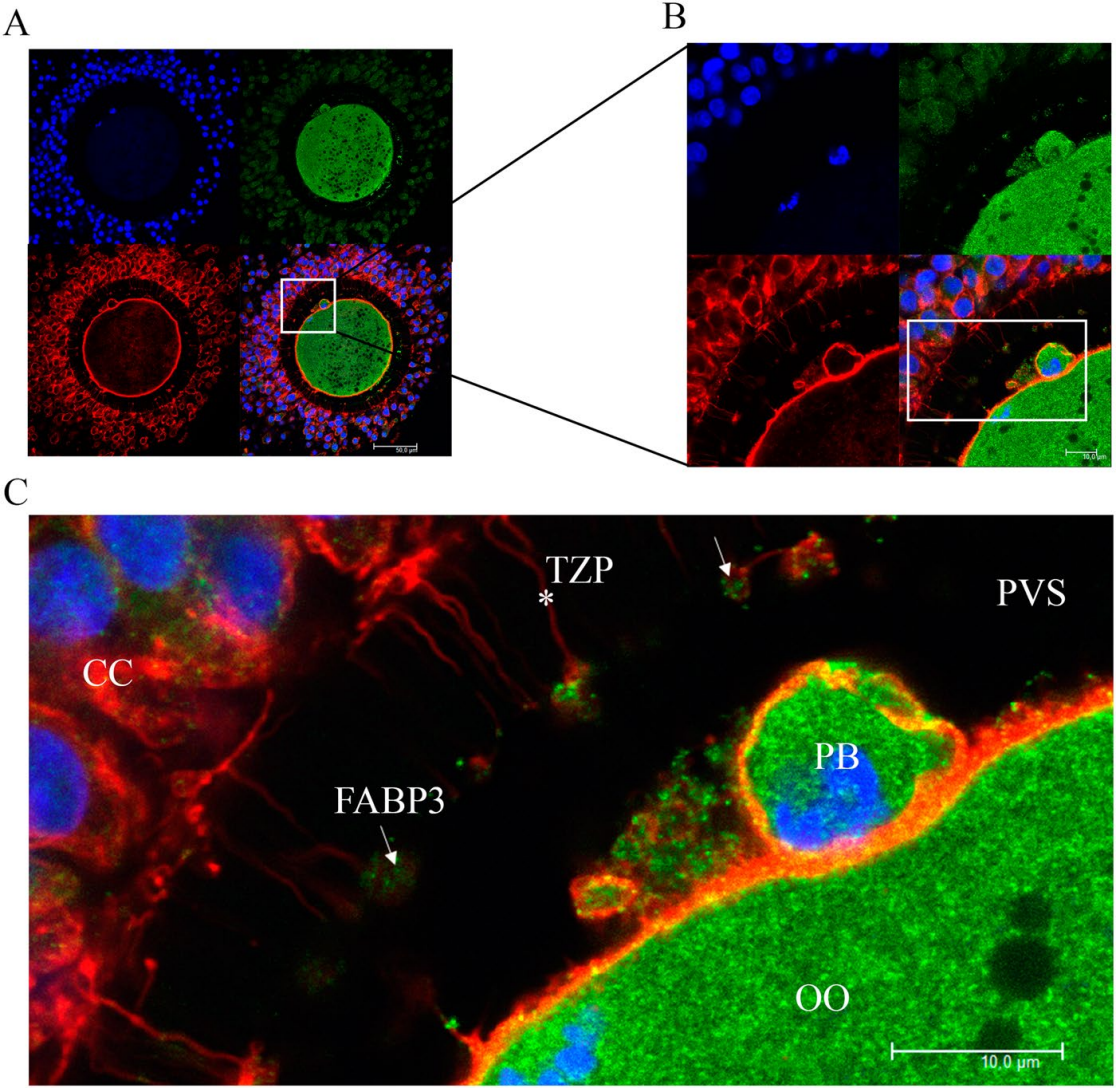
Confocal microscopic analysis of cumulus-oocytes complexes (COCs) after 18 h of IVM. (**A**) Confocal photomicrographs of immunodetection of fatty acid binding protein 3 (FABP3) in tranzonal projections (TZPs) in 18-h matured COCs. (**B**) Photomicrograph showing a detailed view of the COC using a 63x objective and zoomed 3.5x. (**C**) The digital zoom showing the details of the terminal portions of the TZPs disconnected from the ooplasm; FABP3 are immunolocalized at the terminal portion of the TZPs. FABP3 is immunostained using 488 Alexa Fluor (green); TZPs (actin) are stained with Alexa Fluor 647 phalloidin (red); nuclei are stained with DAPI (blue). Photomicrographs were obtained in 63 x objective (**A**) and in 63 x objective zoomed 3.5 x (**B**). Digital zoom (**C**) showing a few FABP3 molecules (arrow) in the terminal portion of TZPs (asterisk) and the perivetellin space (PVS) between oocyte (OO) and zona pellucida. (CC = cumulus cells; PB = polar body). A total of 15 COCs *in vitro*-matured for 18 hours were analysed and showed similar pattern; 6 additional COCs are shown in Supplementary Fig. S7.

**Figure 6:**
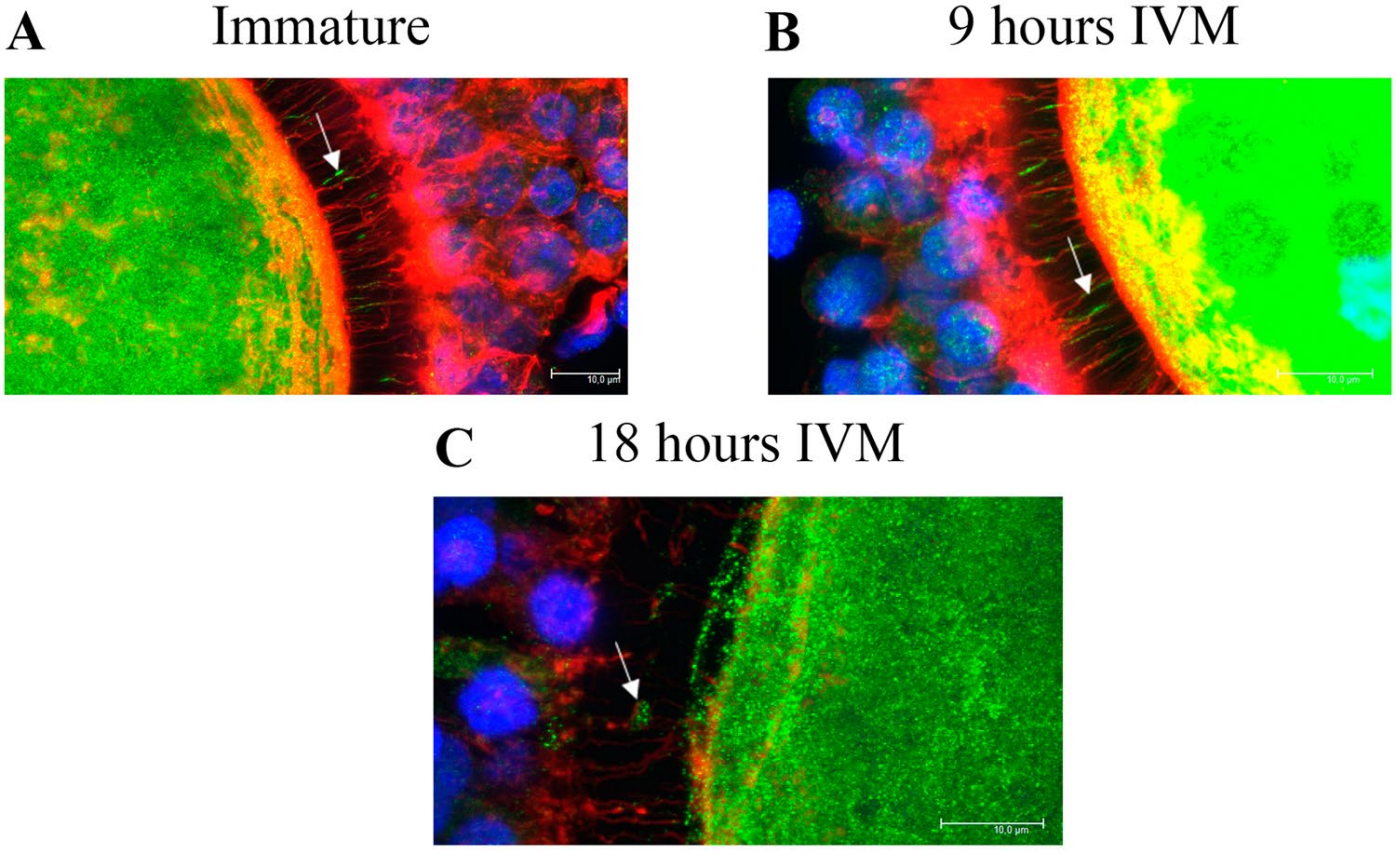
Confocal microscopic analysis of cumulus-oocytes complexes (COCs) at different stages of oocyte maturation (merge of z-stack images) in 63 x objective with digital zoom. (**A**) Photomicrograph showing the localization of fatty acid binding protein 3 (FABP3) and tranzonal projections (TZPs) in immature COCs. (**B**) Photomicrograph showing the localization of FABP3 and TZPs in COCs after 9 h of maturation. (**C**) Photomicrograph showing the localization of FABP3 and TZPs in COCs after 18 h of maturation. Arrows indicate FABP3 immunolocalized in TZPs. FABP3 is immunostained using 488 Alexa Fluor (green); TZPs (actin) are stained with Alexa Fluor 647 phalloidin (red); nuclei are stained with DAPI (blue).

Next, we aimed to investigate whether FABP3 was localized along with the TZPs during IVM. We obtained a series of immunostained images of immature COCs as well as COCs matured *in vitro* for 9 or 18 h. We observed that FABP3 was present within TZPs in immature COCs (Fig. 2 and Supplementary Fig. S3). After 9 h of IVM, the amount of FABP3 present in TZPs increased (Fig. 3 and Supplementary Fig. S4). To verify the presence of FABP3 within TZPs across the zona pellucida (ZP), we subjected oocytes that were either denuded and partially denuded to maturation for 9 h, and then again carried out immunostaining for FABP3 (Fig. 4 and Supplementary Figs S5 and S6). We were unable to detect FABP3 in the ZP of denuded oocytes, suggesting that the presence of TZPs is necessary for the transport of FABP3 between the cumulus and oocytes (Fig. 4a and b). Furthermore, in partially denuded oocytes, we only detected FABP3 along with the TZPs in the ZP (Fig. 4c and d), ruling out the possibility that FABP3 moves between the cumulus and oocytes using other mechanisms. Additionally, because the TZP-mediated transport ceased when oocytes became mature, we investigated the location of FABP3 and TZPs after 18 h of IVM. As expected, we observed that the TZPs were disconnected from the ooplasm and the FABP3 was localized at the terminal portion of the TZPs (Fig. 5 and Supplementary Fig. S7), suggesting that it was derived from cumulus cells. In summary, our results demonstrated that FABP3 was present within TZPs in immature and 9-h matured COCs (Fig. 6a and b). In addition, after 18 h of maturation, when the first polar body was extruded, the TZPs were disconnected from the ooplasm and the FABP3 molecules accumulated at the terminal portion of these projections (Fig. 6c). We also performed a negative control for each immunoreaction: we exposed the samples to the maximum laser potency during confocal microscopy and did not detect any signals in the zona pellucida, ruling out the possibility that our signals are artifacts (Supplemental Fig. S8).

### FABP3 protein levels and lipid droplets increase until up to 9 h in oocytes during IVM

**Figure 7:**
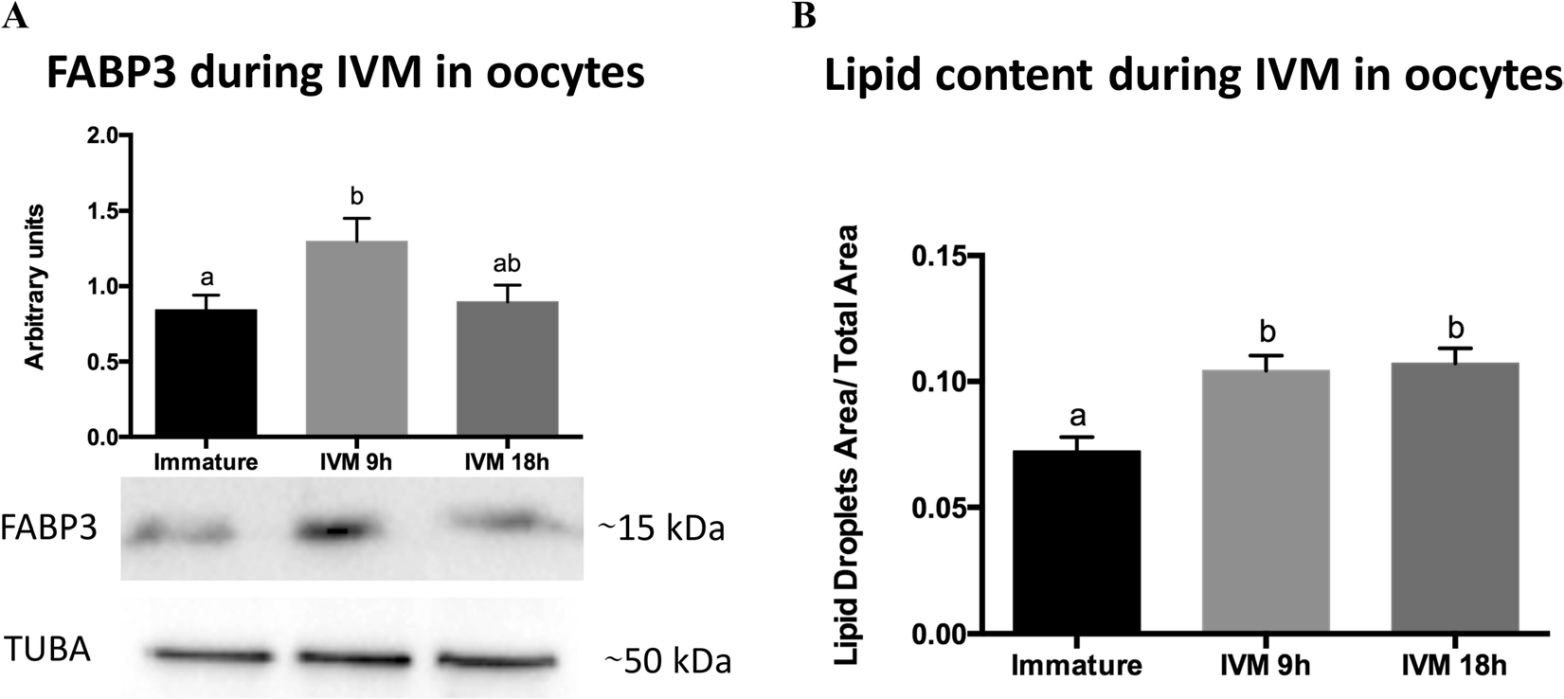
Fatty acid binding protein 3 (FABP3) protein levels and lipid content in oocytes from immature cumulus-oocytes complexes (COCs) and COCs *in vitro*-matured for 9 and 18 h. (**A**) FABP3 protein quantification in oocytes was performed by western blot analysis using x-Tubulin (TUBA) as the normalizer. The full western blot image is shown in Supplementary Fig. S2c. (**B**) Lipid quantification in oocytes. The values for lipid content represent the ratio of the area of lipid droplets to the total oocyte area. Letters above bars in the same graph indicate significant difference (*P* < 0.05). Values are represented as mean ± standard error of the mean.

Since our data suggested the movement of FABP3 from cumulus to oocytes, we expected an increase in the FABP3 protein levels as well as the lipid content in oocytes during IVM. To verify this, we conducted western blot analysis and lipid droplets staining in immature oocytes and in oocytes that underwent IVM for 9 and 18 h. The protein analysis revealed a ~1.55-fold increase in FABP3 levels between immature oocytes and 9-h matured ones, while no difference was observed between 9-h and 18-h matured oocytes (Fig. 7a and Supplementary Fig. S2c). The lipid droplet analysis showed a ~1.44-fold increase in lipid accumulation in 9-h matured oocytes compared to immature ones (Fig. 7b). Interestingly, no differences were observed between 9- and 18-h matured oocytes, suggesting that there was no lipid accumulation during this time. Thus, our results suggested that there was simultaneous accumulation of FABP3 as well as lipids in the oocytes during the first 9 h of IVM.

### The disruption of TZPs in oocytes causes decrease in lipid content

**Figure 8:**
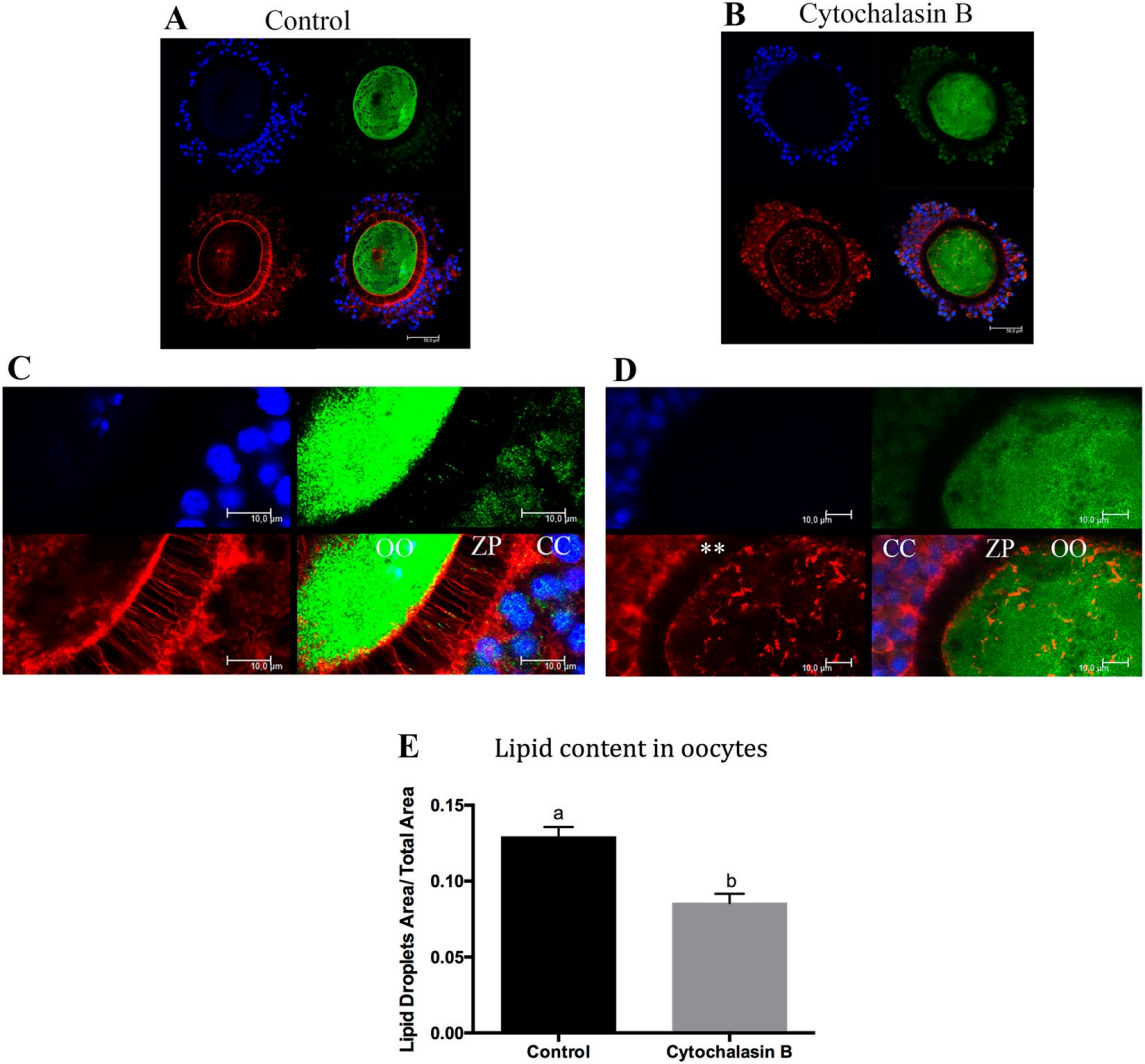
Confocal microscopic analysis of fatty acid binding protein 3 (FABP3) in cumulus-oocytes complexes (COCs) after *in vitro* maturation (IVM) in the absence (control) and presence of cytochalasin B. (**A**) and (**C**) The photomicrographs showing the presence of tranzonal projections (TZPs) and FABP3 in the control group. (**B**) and (**D**) Photomicrographs showing the lack of most parts of TZPs and FABP3 within the zona pellucida. FABP3 was immunostained using 488 Alexa Fluor (green); TZPs (actin) are stained with Alexa Fluor 647 phalloidin (red); nuclei are stained with DAPI (blue). Photomicrographs were obtained in 63x objective (**A** and **C**); and in 63 x objective zoomed 3.5 x (**B** and **D**). CC = cumulus cells; ZP = zona pellucida; OO = oocyte. **Indicate the disassembly and blocking of TZP formation by cytochalasin B. A total of 10 control COCs and 10 cytochalasin treated COCs *in vitro*-matured for 9 hours were analysed and showed similar pattern; 6 additional control COCs and 6 additional cytochalasin B treated COCs are shown in Supplementary Fig. S10. (**E**) Lipid content in oocytes from COCs after IVM for 9 h in the absence (control) or presence of cytochalasin B. Lipid quantification was performed by fluorescence confocal microscopy to detect lipid droplets. Representative control and cytochalasin B-treated oocytes stained with BODIPY 493/503 are shown in Supplementary Fig. S11. The values for lipid content represent the ratio of the area of lipid droplets to the total oocyte area. Letters above bars indicate significant differences (*P* *<* 0.05). Values are represented as mean ± standard error of the mean.

Because our results suggested that lipids are transported from cumulus cells to oocytes in a TZP-dependent manner, we decided to disrupt the TZPs to further clarify its role in lipid accumulation. To this end, we utilized cytochalasin B, an agent that blocks the polymerization of actin filaments and thus disrupts the TZPs. We exposed the COCs to cytochalasin B during the first 9 h of IVM to verify whether the lipid content was affected. Our results demonstrated that most of the TZPs were disrupted by cytochalasin B during IVM (Figs 8a–d and Supplementary Fig. S10). The total lipid content in oocytes that matured in the presence of cytochalasin B was lower than that in the control oocytes (Fig. 8e), indicating that functional TZPs are necessary to drive lipid accumulation.

## Discussion

The increased lipid accumulation in *in vitro*-produced embryos is one of the major disadvantages of IVP, causing impaired cryotolerance and low pregnancy rates^1, 2^. It was demonstrated that this accumulation is one of the consequences of IVM in species such as bovine and mouse, and in bovine is caused by exposure of the gametes to a non-physiological environment^3, 6^. In the present study, we proposed that the excessive lipid accumulation in *in vitro*-matured oocytes is mediated by the dysregulated transport of a fatty acid lipid-carrier protein named FABP3 through TZPs. We demonstrated that the *in vitro* system caused an increase in FABP3 levels. Additionally, an increase in lipid content during IVM was associated with the increase in FABP3 protein levels in *in vitro*-matured cumulus cells. By comparing immature oocytes with 9- and 18-h matured ones, we observed the presence of FABP3 within TZPs during IVM as well as dynamic changes in FABP3 protein levels and lipid levels in oocytes during the maturation process. In addition, we showed that the disruption of TZPs in oocytes during the first 9 h of maturation decreased the lipid accumulation. Thus, our results demonstrated, for the first time, a probable mechanism for the transport of fatty acids from cumulus cells to the oocytes, which may be responsible for the lipid accumulation in oocytes.

Lipid transportation from the medium to the cells or among the cells in *in vitro* systems has been extensively investigated^3, 4, 23^ as a possible cause of lipids accumulation within *in vitro*-produced embryos. Many studies have reported that embryos cultured in media containing fetal bovine serum acquired more lipids and were less cryotolerant than those grown without serum^4, 5, 23^. Based on that, a relationship between the lipid content of the maturation medium and the elevated lipid levels observed in oocytes after IVM has been established. However, the mechanisms mediating lipid transport within COCs during IVM have never been identified and remains unknown.

We observed that FABP3 was localized along the TZPs during IVM, suggesting that FABP3 molecules that originated from the cumulus may transport fatty acids from these cells to the oocytes, and eventually end up in the oocytes. TZPs, also known as cumulus cells process endings (CCPEs)^24, 25^, are able to transport molecules <1 kDa to the oocyte by GAP junction located at the final portion of TZPs^26, 27^ or by the recently proposed traffic of extracellular vesicles into the cleft between the end of TZPs and the oolema^13, 28^. Based on the molecular weight of FABP3 (>1 kDa), the transport to oocytes mediated by vesicles is the probable mechanism. This supposition finds a parallel in the reports of transport of large molecules such as mRNA from cumulus cells to the oocytes mediated by TZPs within COCs^13, 29^. Additionally, FABP5 as well as other members of this protein family such as FABP4 and FABP3 were described in extracellular vesicles from different body fluids^17, 30–34^. Thus, based on the presence of extracellular vesicles in the cleft between TZPs and the ooplasm^13^, a reasonable hypothesis is that FABP3s are transported to the oocyte by extracellular vesicles secreted by cumulus cells.

The family of FABP proteins was for a long time associated with intracellular fatty acid transport, mostly to the mitochondria, where β-oxidation takes place. However, recently, FABP proteins were discovered to possess extracellular functions as well, carrying lipids between tissues^35^. Additionally, studies have reported that extracellular FABP proteins were altered in metabolism-associated diseases^17, 30, 36–41^. Cardiac FABP, also known as H-FABP or FABP3, was first observed in reproductive tissues such as ovary and placenta, where it performed both intra- and extracellular functions^17, 20^. Based on the current literature, FABP3 and FABP5 are the only FABPs expressed in bovine COCs, but only FABP3 increased during IVM in bovine oocytes, suggesting a possible role in lipid accumulation^22, 42^.

Exposure of muscle cells to high levels of fatty acids both *in vivo* and *in vitro* induced the expression of FABP3^43, 44^. Because we observed an increased lipid accumulation in COCs, we decided to investigate whether FABP3 in cumulus cells were altered after IVM. We demonstrated an increase in lipid content as well as FABP3 transcript and protein levels in cumulus cells undergoing IVM. We also observed an increase in lipid content in oocytes after exposure to an *in vitro* environment, suggesting that FABP3 might be involved in extracellular fatty acid transport as well, instead of only in the canonical intracellular function. Additionally, *in vivo*-matured cumulus cells did not show any increase in the levels of FABP3 compared to immature cumulus cells, indicating that the *in vitro* system caused the dysregulation of lipid transport. Altogether, our data suggested that the mechanism leading to the increased lipid content observed in oocytes after IVM was mediated by FABP3.

From the results of our immunolocalization experiments we verified the relationship between FABP3 and lipid content in oocytes during the initial stage of IVM, by observing the co-localization of FABP3 and TZPs during the first 9 h of IVM. Therefore, the increased FABP3 levels and lipid content observed at this stage might have been caused by the transport of FABP3 through TZPs. We also demonstrated that after 18 h of IVM, the number of TZPs near the ooplasm decreased drastically, and that FABP3 molecules were localized at the terminal portion of the projections. The fact that FABP3 migrated only until the terminal portion of the TZPs and accumulated there, suggested that traffic occurs only from cumulus cells to oocytes and not in the opposite direction. The FABP3 levels and lipid content in the oocytes remained stable from 9 h to 18 h of IVM. Thus, the increase in FABP3 and lipids seemed to be dependent on the presence of active TZPs.

The necessity of functional TZPs to allow for lipid accumulation in oocytes is supported by the literature. The exposure of COCs to high levels of free fatty acid during the last 6 hours of *in vitro* maturation (considering a 23 h IVM) resulted in lipid accumulation in cumulus cells, but not into oocytes^45^. Therefore, in moment where TZPs are no longer functional, the traffic of lipid from cumulus to oocytes seems to be impaired. Thus, previous work demonstrated increased accumulation of lipids within the cumulus cells, following the exposure of the COCs to rich-lipids environment^45^; however, here we demonstrated a possible fatty acid transport mechanism from cumulus cells to oocyte during IVM.

To verify whether TZPs are involved in fatty acid transport from cumulus cells to oocytes, we disrupted TZPs using cytochalasin B during the first 9 h of IVM. Initially, we observed that cytochalasin B-treated oocytes lacked most of the TZPs because of the disruption and that FABP3 was not present within the ZP. Therefore, our treatment disrupted the TZPs and blocked the transport of FABP3 from cumulus cells through the ZP. Additionally, we demonstrated a decrease in lipid content in the cytochalasin B-treated oocytes compared to the non-treated controls. Hence, disruption of TZPs decreased lipid accumulation in oocytes, impairing fatty acid transport from cumulus cells to oocytes, thus supporting our initial speculation.

The results of this work shed light on the effects of exposing gametes to *in vitro* culture conditions during ARTs. The reasons why *in vitro* maturation triggers lipid accumulation and FABP3 upregulation are still elusive. Lipid-rich *in vitro* environment do not seen be responsible, as previously discussed^3^. Metabolic dysregulation (probably of cumulus cells), including abnormal energy metabolism and abnormal triacylglycerol synthesis, are the candidate pathways to trigger lipid accumulation. However, these hypotheses are yet to be confirmed. The results of this work also shed light on fertility impairment in women suffering from metabolic disorders. It is well known that women who are overweight and/or suffering from metabolic syndromes have several fertility problems associated with high lipid content in the blood, follicular fluids, and oocytes. This elevated level of lipid may trigger endoplasmic reticulum stress and consequently apoptosis in COCs due to a lipotoxicity response, leading to the decreased fertilization rates observed in these women^9–11^. However, the mechanism behind this lipid accumulation remains unknown. Recent studies have identified high levels of FABP4 in the blood and tissues of patients with obesity^17^. Likewise, FABP3 levels were found to be increased in patients with metabolic syndrome^46^. Even though these types of pathologies are increasingly becoming frequent among women and are amongst the major causes of modern fertility-related problems, we still do not understand the molecular mechanism underlying these issues.

Our results demonstrated that lipid accumulation during IVM was dependent upon the functional communication between cumulus cells and oocytes. We also suggested a possible mechanism for fatty acid transport from cumulus cells to oocytes *via* FABP3 and TZPs. We demonstrated the dysregulation of FABP3 synthesis and its accumulation in oocytes during IVM, concomitant with the lipid accumulation. We suggested that an increase of lipid accumulation in cumulus cells during IVM could lead to an increase of FABP3 expression levels to transport those fatty acids. Thus, this dysregulation in cumulus cells could lead to increased lipid accumulation in oocytes during IVM. Further experiments are necessary to verify this new mechanism of lipid accumulation in oocytes during IVM. Our data strongly suggested that fatty acid transport within COCs is mediated by FABP3 and that FABP3 may play a role in fertility issues. Finally, a better understanding of this mechanism of transport will help in the development of tools to modulate lipid accumulation in oocytes during IVM; this could have great implications for ARTs in animals and humans and for fertility treatments for women suffering from metabolic disorders.

## Methods

All reagents were purchased from Sigma Chemical Co. (St. Louis, MO, USA) unless otherwise indicated. The Bioethical Committee of the FZEA - University of Sao Paulo, Pirassununga, SP, Brazil has approved this study under the protocol number 14.1.675.74.7. We adopted the International Guiding Principles for Biomedical Research Involving Animals (Society for the Study of Reproduction) as well.

### The lipid content and FABP3 levels during *in vivo* and IVM

#### Recovery of immature, *in vitro*-, and *in vivo*-matured oocytes

In order to compare immature, *in vitro*-, and *in vivo*-matured oocytes and cumulus cells, Nellore (*Bos indicus*) COCs were obtained from slaughterhouses or from live cows by the ovum pick-up (OPU) method. To obtain oocytes for IVM, we performed follicular aspiration of the Nellore cow-ovaries obtained post-mortem. To obtain *in vivo*-matured oocytes, we synchronized the ovarian follicular wave of cyclic Nellore cows and then subjected them to follicular superstimulation and OPU. The detailed protocols are presented in the supplementary materials and methods (Supplementary File).

#### Confocal microscopic analysis of lipid content in oocytes

Lipid quantifications analyses were performed by staining the lipid droplets with a dye specific for neutral lipids, – BODIPY 493/503 (Molecular Probes, Eugene, OR, USA), followed by confocal microscopy imaging. We utilized denuded oocytes from the three groups (49 immature, 46 *in vivo-*matured, and 45 *in vitro-*matured oocytes, obtained from 12 biological replicates) for confocal analysis. The denuded oocytes were fixed, permeabilized, and stained following the manufacturer’s instructions. The oocytes were then analyzed using the LSM 710 confocal microscope (Carl Zeiss, Oberkochen, Germany) at 63X magnitude in oil with an Argon 488 laser. One image per oocyte was captured choosing the largest diameter section and later analyzed with the ImageJ program (NIH; http://rsb.info.nih.gov/ij/). The ratio of the lipid droplet area to the total oocyte area was determined and analyzed with ImageJ, as previously described^3^. Briefly, lipid content in oocytes was visualized using fluorescence confocal microscopy and quantified based on the fraction of area occupied by lipid droplets within the ooplasm. To obtain area of lipid droplets, we transformed the original image to 8 bits image; and then we determined the lipid droplets area with a plugin named “nucleus counter” (detailed in Supplemental Fig. S9).

#### Quantification of PLIN2 and FABP3 in cumulus cells by western blot

Lipid content in cumulus cells was determined based on the amount of PLIN2, a protein present in lipid droplets and commonly used as a marker for lipid accumulation studies^6, 11, 47, 48^. To evaluate the levels of PLIN2 and FABP3 in cumulus cells, we performed western blot analysis for immature, *in vivo*-, and *in vitro*-matured cumulus cells. The proteins utilized in these analyses were from the same pool of samples that we used for the gene expression studies as we had stored the organic phase left over after RNA extraction using TRIzol reagent. The protein was isolated from the organic phase of the TRIzol reagent according to the manufacturer’s instruction. To achieve the protein quantity required for western blot analysis, samples from the same biological group were pooled and analyzed in three technical replicates. A total of 50 μg of protein was loaded and resolved using a 10%-SDS- PAGE commercial kit, Mini-Protean TGX Gels (456–1033, Bio-RAD, Hercules, CA, USA). Electrophoresis was performed at 100 V for 70 min, and the proteins were transferred onto a polyvinylidene difluoride PVDF membrane using a Trans-Blot Turbo Transfer Pack (170–4156, Bio-RAD) in a semidry transfer apparatus, Trans-Blot Turbo, using a “mini TGX program” (3 min at 25 V constant). The membranes were then placed in a blocking buffer that had 5% bovine serum albumin (BSA) in tris buffered saline with tween (TBST) for 1 h at room temperature. Next, the membranes were incubated overnight with the primary antibody for PLIN2, anti-ADRP rabbit antibody (1:2000, SC32888, Santa Cruz Biotechnology, Dallas, Texas, USA), at 4 °C. Following overnight incubation, the membranes were washed thrice for 5 min with TBST and then incubated with the secondary antibody, anti-rabbit conjugated with HRP antibody (1:2000, A0545, Sigma), for 1 h at room temperature. To detect FABP3, we used an anti-cardiac FABP rabbit antibody (1:2000, AB45966, Abcam) and the secondary anti-rabbit antibody conjugated with HRP (1:4000; A0545, Sigma). The membranes were again washed thrice in 1x TBST for 5 min. For detection, the membranes were subjected to a chemiluminescent reaction using the reagent ECL Plus Prime Western Blotting Detection System solution (Amersham™, Buckinghamshire, UK). Imaging and band density analyses were performed using ChemiDoc MP Image System (Bio-Rad,) and the software Image Lab 5.1, respectively. The relative amounts of PLIN2 were normalized using the anti-histone H3 rabbit antibody (1:2000, H0164, Sigma) blotted in the same membrane. The relative amounts of FABP3 were normalized using the anti-x-Tubulin mouse antibody (1:2000, T9026, Sigma) and an anti-mouse antibody (1:2000, #70765, Cell Signaling) as endogenous controls.

#### Relative mRNA analysis

To investigate the levels of FABP3 transcript, we extracted mRNA from five pools, each of then containing 20 immatures and *in vivo*- and *in vitro*-matured denuded oocytes (in MII phase) that were collected from 12 biological replicates of *in vivo* and *in vitro* maturation. For the analysis of cumulus cells, we used 8 different pools of cumulus cells retrieved from 20 immature, 10 *in vivo*-, and 10 *in vitro*-matured COCs, representing the same biological replicates. The total RNA was extracted using the TRIzol reagent (ThermoFisher, Waltham, Massachusetts, USA) and the miRNeasy Kit (Qiagen, Valencia, CA, USA). Total RNA was quantified using the NanoDrop 2000 (Thermo Scientific) and the quality was estimated using the 260/280 ratio. Reverse transcription of mRNA was performed using the High-Capacity cDNA Reverse Transcription kit (Applied Biosystems, Foster City, CA, USA) according to manufacturer’s instructions. Approximately 100 ng of the total RNA was incubated with 10x RT Buffer, 25x dNTP mix (100 mM), 10x RT Random Primers, and nuclease-free water at 25 °C for 10 min and at 37 °C for 120 min, followed by 5 min at 85 °C to stop the reaction. For real-time quantitative reverse transcription-PCR (RT-qPCR), 10 ng of RNA at the initial concentration was used for each gene. RT-qPCRs were performed in duplicate using 1 μl of cDNA of each gene and 75 nM of primers using the Power SYBR® Green PCR Master Mix kit (Applied Biosystems) in the QuantStudio 6 Flex PCR System (Applied Biosystems). The PCR cycle conditions were as follows: 95 °C for 10 min, 40 cycles of 95 °C for 15 s, and 60 °C for 60 s. The normalized Ct levels for *FABP3* were obtained by the subtracting the Ct of the target gene using the geometric means of three reference genes (*PPIA*, *SDHA*, and *YWAHZ)*. These endogenous genes have been demonstrated to be stably expressed and suitable for use as normalizers in gene expression studies of bovine oocytes^49^. The primers were designed based on GenBank sequences (Supplementary Table S1) and tested for efficiency by running a standard curve; the specificity was confirmed by sequencing the amplicon.

### Immunolocalization of FABP3 within TZPs and quantification of FABP3 levels and lipid content in oocytes during IVM

Through the *in vivo* and IVM studies, we aimed to understand FABP3 and lipid behavior during IVM. First, we performed immunodetection of FABP3 in TZPs in immature and 9- and 18-h matured COCs in order to evaluate changes in the location of this protein. Subsequently, we studied FABP3 levels and the lipid content during IVM to understand the role of FABP3 in mediating fatty acid transport to oocytes. For these experiments, we collected grade I and II COCs from 3–6-mm follicles by postmortem follicular aspiration of bovine ovaries. The COCs had undergone IVM as described previously, and were then removed from the maturation media for analysis at 9 and 18 h after the beginning of IVM.

#### Immunodetection of FABP3 in TZPs within COC

FABP3 were detected in TZPs by immunofluorescence analysis after staining the actin filaments in order to visualize the TZPs. We stained immature COCs (n = 12), as well as COCs *in vitro-*matured for 9 (n = 12) and 18 h (n = 15). We also analyzed 10 denuded and 11 partial denuded oocytes submitted to IVM for 9 hours. The COCs assigned for immunodetection of FABP3 were obtained from three biological replicates. The COCs were fixed with 4% paraformaldehyde diluted in phosphate buffered saline (PBS) with 0.1% polyvinylpyrrolidone (PBS-PVP) for 12 minutes. COCs were then permeabilized for 20 min in PBS-PVP with 1% Triton X-100. After blocking of non-specific binding sites with 5% BSA in PBS for 1 h, COCs were incubated overnight at 4 °C with the primary rabbit antibody anti-FABP3 (1:200, SC15974R, Santa Cruz Biotechnology) and 1% BSA in PBS for FABP3 detection. Following incubation, the samples were washed in PBS-PVP and incubated with the secondary antibody, anti-rabbit conjugated with Alexa 488 (A11008, Life Technologies, Thermo Fisher) diluted at 1:200. After incubation, the samples were washed ten times in PBS-PVP to remove all non-specifically bound secondary antibodies. To label the TZPs, COCs were stained with 165 nM of Alexa Fluor 647 phalloidin (A22287, Life Technologies, Thermo Fisher), an actin filament stain, for 30 minutes. Oocytes were mounted on glass slides with coverslips using Prolong Antifade with DAPI reagent (Life Technologies, Thermo Fisher). For capturing images, a SP5 confocal microscope (Leica, Wetzlar, Germany) was used with Diode 405 nm, Argon 488 nm, and HeNe 633 nm adjusted to each probe. All images were captured by sequential acquisition, in 63x magnification in oil, and in 3.5x magnification for detecting TZPs. For the negative control, we omitted the incubation with primary antibody and acquired the images utilizing maximum laser potency to rule out the possibility of artifacts.

#### Confocal microscopic analysis of lipid content in oocytes

To evaluate a possible relationship between FABP3 transport and lipid accumulation in oocytes, we carried out a time course experiment on immature oocytes and oocytes *in vitro*-matured for 9 and 18 h. We collected a total of 27 immature oocytes, 31 oocytes after 9 h of IVM, and 28 oocytes after 18 h of IVM. We then quantified the lipid content in these oocytes as described in the previous section.

#### Quantification of FABP3 in oocytes by western blot

Western blot analyses were performed in 5 pools with 50 denuded oocytes from each group. The pools of oocytes were directly lysed in 7.5 μL of RIPA buffer, mixed with 2.5 μL Laemmli buffer (4x), boiled at 98 °C for 5 min, and then loaded onto a SDS-PAGE gel. All the other steps were performed as described in the previous sections.

### Disruption of TZPs during IVM using cytochalasin B

To investigate the effects of blocking TZPs on lipid accumulation, we disrupted the TZPs during oocyte maturation using cytochalasin B, a potent fungal toxin that hampers actin polymerization. We treated the oocytes with cytochalasin B (15.7 μM) in the maturation medium for 9 h in order to disrupt the TZPs. To observe the effects of TZP-disruption, we stained the actin filaments and FABP3 in 10 treated COCs and 10 untreated (control) COCs as described previously. This experiment was conducted in three biological replicates. The reduction in TZPs after cytochalasin B treatment was confirmed using an immunofluorescence analysis. Additionally, we quantified the lipid content using fluorimetric analysis of oocytes after 9 h of IVM in the presence (n = 31) and absence (n = 31) of cytochalasin B.

### Statistical Analysis

The mRNA expression data were analyzed using the one-way ANOVA and the averages were compared using Tukey’s test. The data from the western blot were subjected to logarithmic transformation before ANOVA and Tukey’s test. These analyses were performed in SAS software. The lipid accumulation data were compared using the non-parametric Kruskal Wallis test followed by Dunn’s multiple comparison test, and were performed using PRISM from Graphpad software. A 5% level of significance was adopted for all tests. Data were presented as mean ± standard error of the mean.

## Electronic supplementary material

Supplementary files
