# Supplementary material for: Fatty Acid Binding Protein 3 And Transzonal Projections Are Involved In Lipid Accumulation During In Vitro Maturation Of Bovine Oocytes
Source: Sci Rep. 2017 Jun 1;7:2645. doi: 10.1038/s41598-017-02467-9 (PMC5453981; doi:10.1038/s41598-017-02467-9)
Supplement: Supplementary file 1 — Supplementary files [file 41598_2017_2467_MOESM1_ESM.pdf]

Title: FATTY ACID BINDING PROTEIN 3 AND TRANSZONAL PROJECTIONS ARE INVOLVED IN LIPID ACCUMULATION DURING *IN VITRO* MATURATION OF BOVINE OOCYTES

Authors: Maite del Collado; Juliano Coelho da Silveira; Juliano Rodrigues Sangalli; Gabriella Mamede Andrade; Letícia Rabello da Silva Sousa; Luciano Andrade Silva; Flavio Vieira Meirelles; Felipe Perecin

SUPPLEMENTARY FIGURES

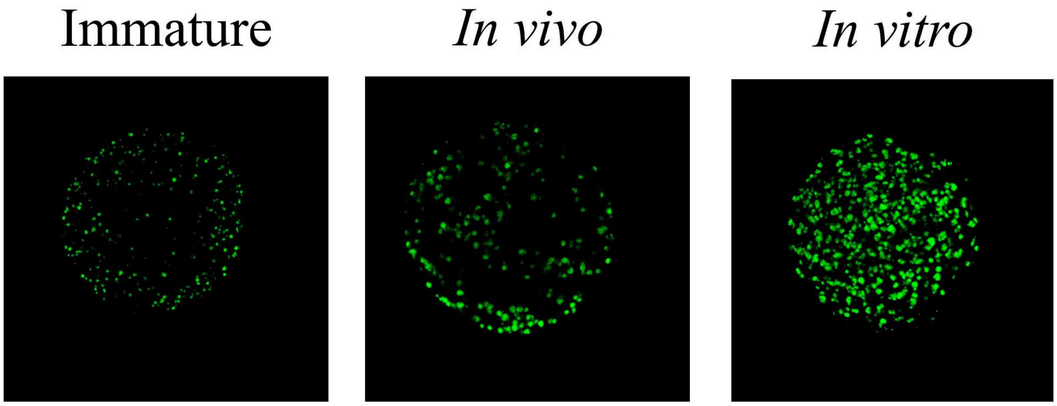

Supplementary Figure S1. Representative confocal photomicrographs of immature, *in vivo*-, and *in vitro*-matured oocytes stained for lipid quantification with BODIPY 493/503 (Molecular Probes, Eugene, OR, USA). Images were captured in LSM 710 confocal microscope (Carl Zeiss) using 63 x objective.

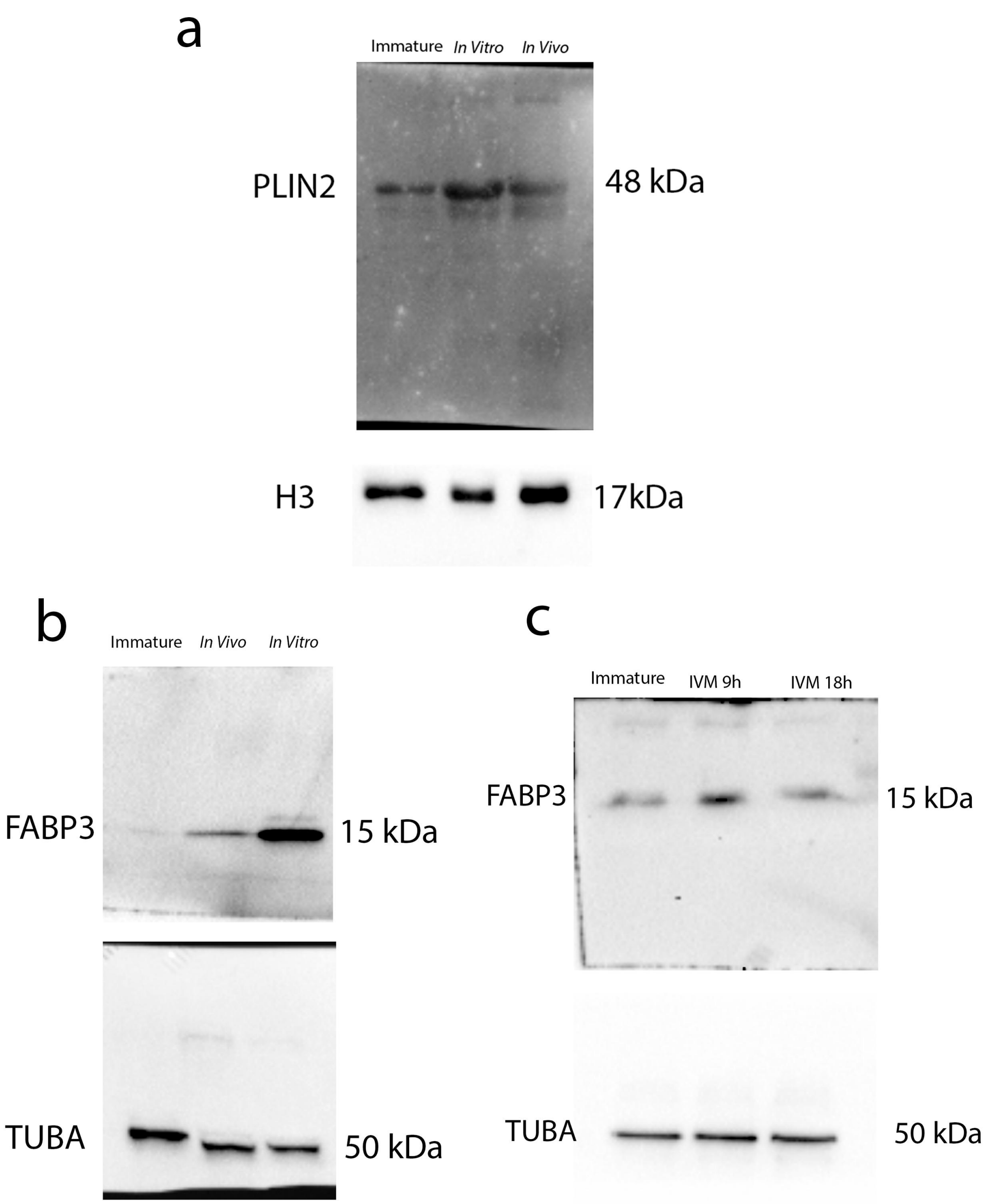

Supplementary Figure S2. Full imagens of western blot membranes. (a) western blot analysis of perilipin 2 (PLIN2) using histone 3 (H3) as normalizer in immature, *in vitro*- and *in vivo*- maturated cumulus cells; (b) western blot analysis of Fatty Acid Binding Protein 3 (FABP3) using Tubulin (TUBA) as normalizer in immature, *in vitro*- and *in vivo*- maturated cumulus cells; (c) western blot analysis of FABP3 and TUBA in immature, *in vitro*- and *in vivo*- maturated oocytes.

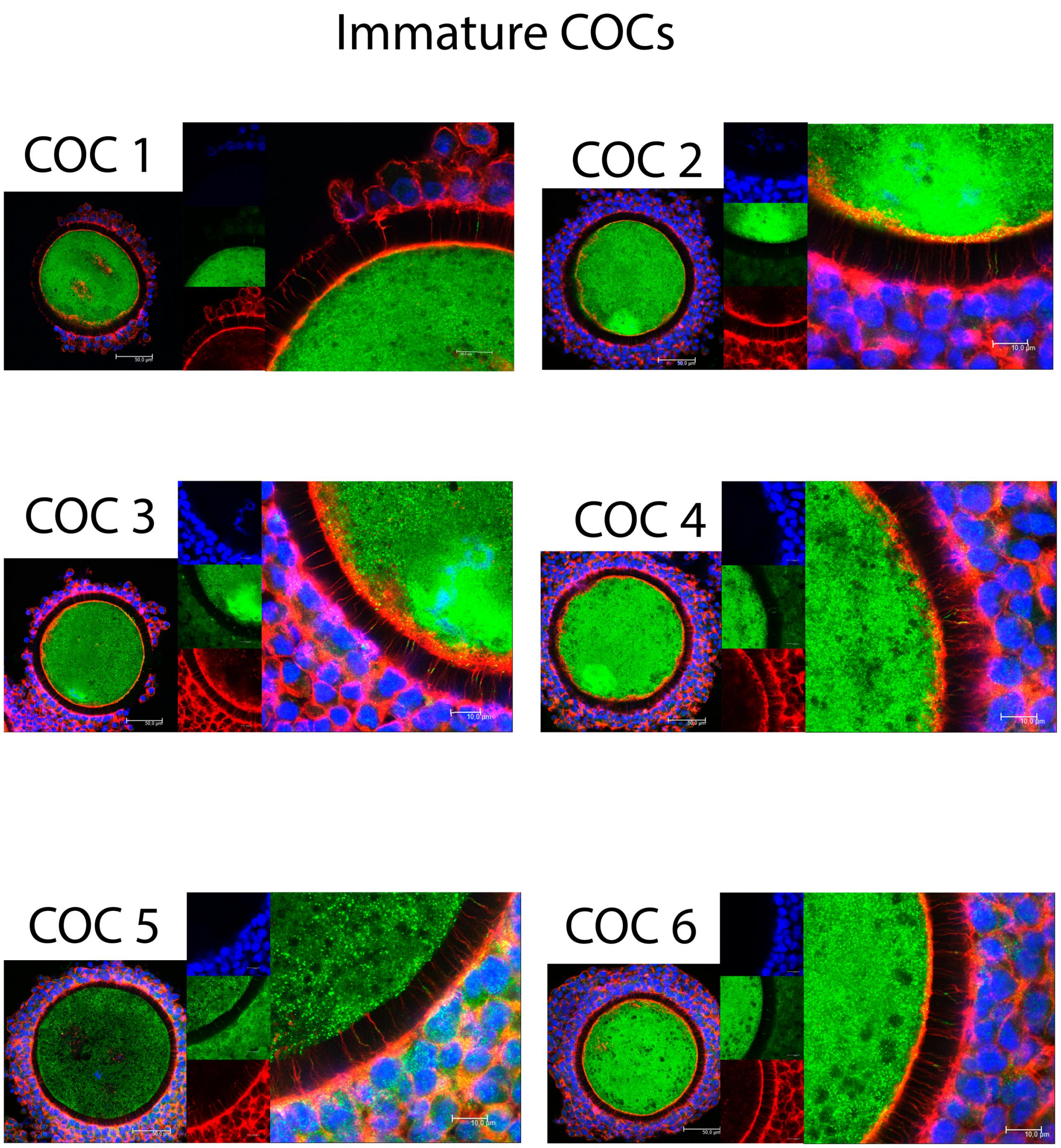

Supplementary Figure S3. Supplementary confocal photomicrographs of 6 immature cumulus-oocyte complexes (COCs). For each COC, the image at left was obtained in 63 x objective and is the merged z-stacked captures. The three images at the center were obtained in 63 x objective zoomed 3.5 x. From top to bottom these three images are stained to visualize: nuclei stained with DAPI (blue – the image at the top); FABP3 immunostained using 488 Alexa Fluor (green – the image at the middle); and TZPs (actin) stained with

Alexa Fluor 647 phalloidin (red – the image at the bottom). The large image at right is the digital zoom of merged z-stacked captures.

COCs matured for 9 hours

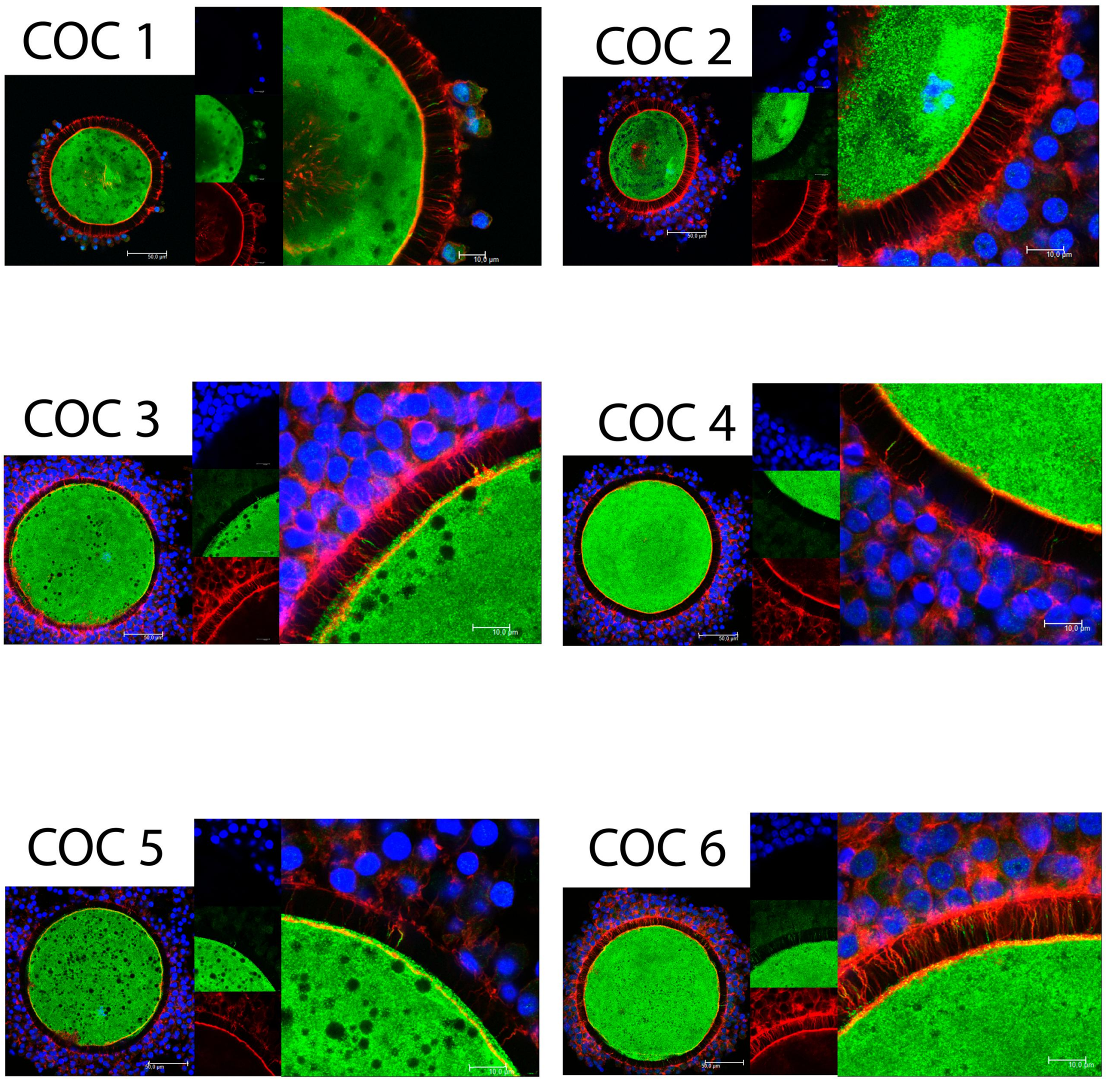

Supplementary Figure S4. Supplementary confocal photomicrographs of 6 cumulus-oocyte complexes (COCs) *in vitro*-matured for 9 hours. For each COC, the image at left was obtained in 63 x objective and is the merged z-stacked captures. The three images at the center were obtained in 63 x objective zoomed 3.5 x. From top to bottom these three images are stained to visualize: nuclei stained with DAPI (blue – the image at the top); FABP3 immunostained using 488 Alexa Fluor (green – the image at the middle); and TZPs

(actin) stained with Alexa Fluor 647 phalloidin (red – the image at the bottom). The large image at right is the digital zoom of merged z-stacked captures.

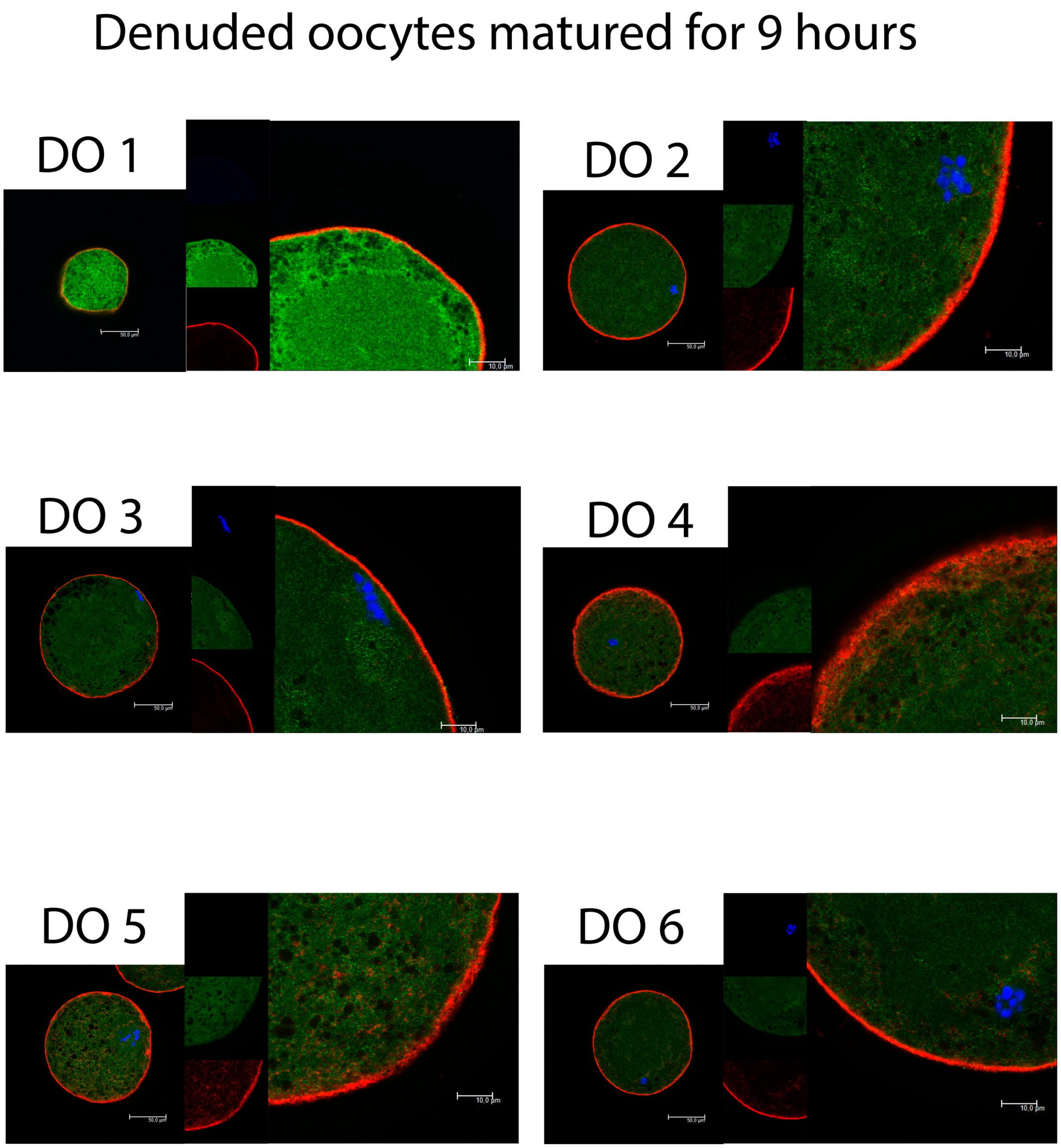

Supplementary Figure S5. Supplementary confocal photomicrographs of 6 denuded-oocytes *in vitro*-matured for 9 hours. For each oocyte, the image at left was obtained in 63 x objective and is the merged z-stacked captures. The three images at the center were obtained in 63 x objective zoomed 3.5 x. From top to bottom these three images are stained to visualize: nuclei stained with DAPI (blue – the image at the top); FABP3 immunostained using 488 Alexa Fluor (green – the image at the middle); and TZPs (actin) stained with

Alexa Fluor 647 phalloidin (red – the image at the bottom). The large image at right is the digital zoom of merged z-stacked captures.

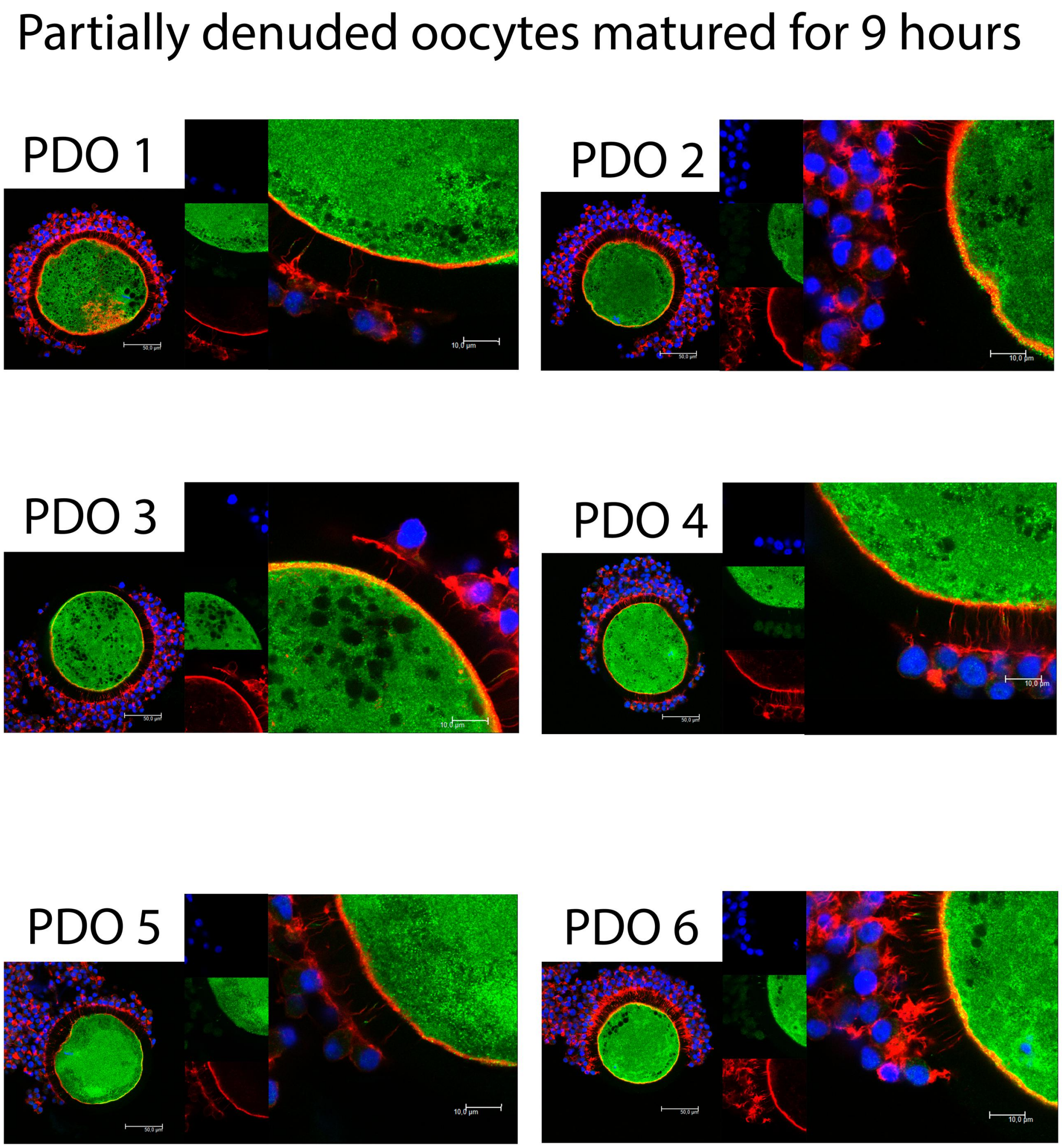

Supplementary Figure S6. Supplementary confocal photomicrographs of 6 partially denuded-oocytes *in vitro*-matured for 9 hours. For each oocyte, the image at left was obtained in 63 x objective and is the merged z-stacked captures. The three images at the center were obtained in 63 x objective zoomed 3.5 x. From top to bottom these three images are stained to visualize: nuclei stained with DAPI (blue – the image at the top); FABP3 immunostained using 488 Alexa Fluor (green – the image at the middle); and TZPs

(actin) stained with Alexa Fluor 647 phalloidin (red – the image at the bottom). The large image at right is the digital zoom of merged z-stacked captures.

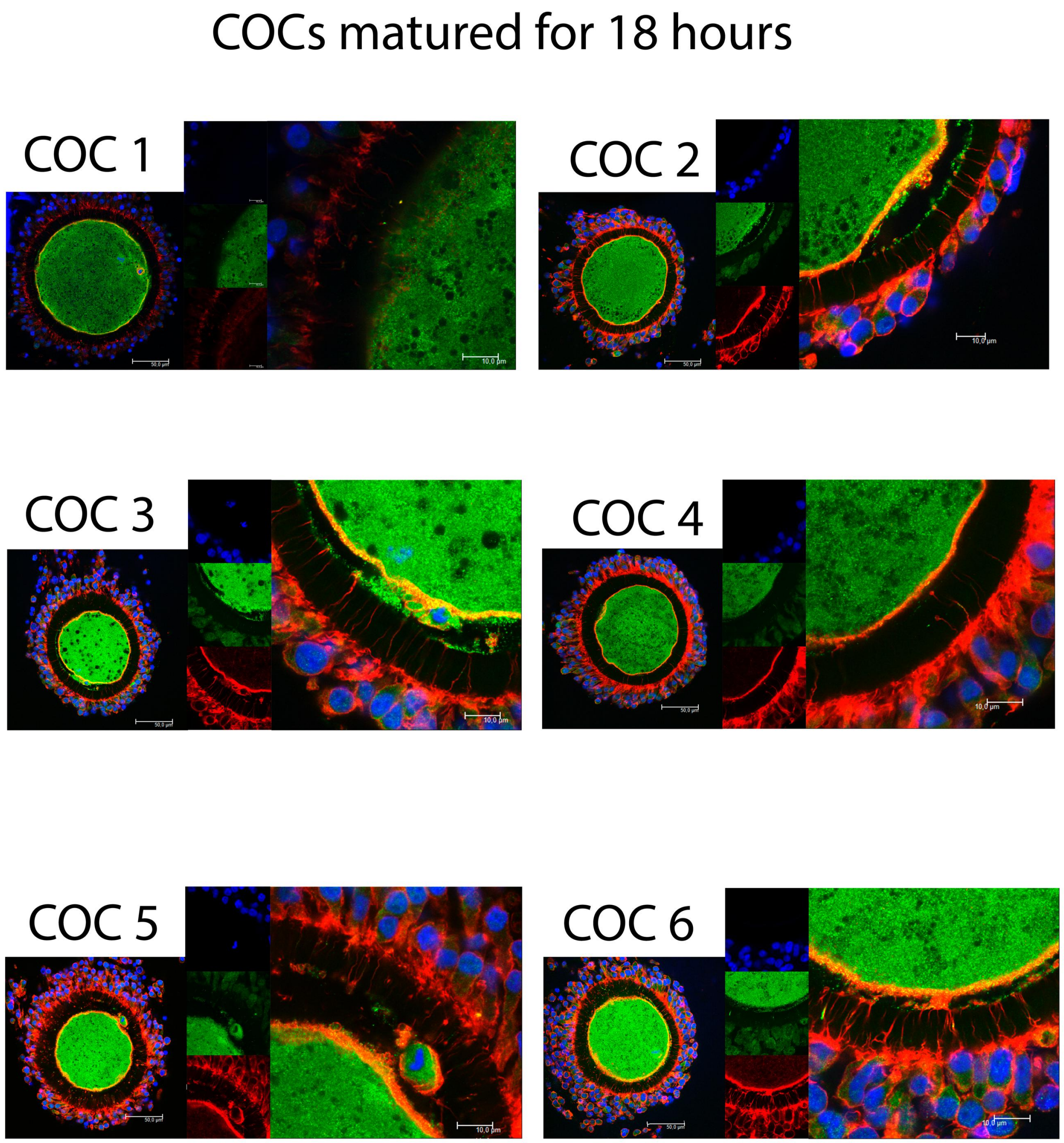

Supplementary Figure S7. Supplementary confocal photomicrographs of 6 cumulus-oocyte complexes (COCs) *in vitro*-matured for 18 hours. For each COC, the image at left was obtained in 63 x objective and is the merged z-stacked captures. The three images at the center were obtained in 63 x objective zoomed 3.5 x. From top to bottom these three images are stained to visualize: nuclei stained with DAPI (blue – the image at the top); FABP3 immunostained using 488 Alexa Fluor (green – the image at the middle); and TZPs

(actin) stained with Alexa Fluor 647 phalloidin (red – the image at the bottom). The large image at right is the digital zoom of merged z-stacked captures.

FABP3 inmunodetection negative control

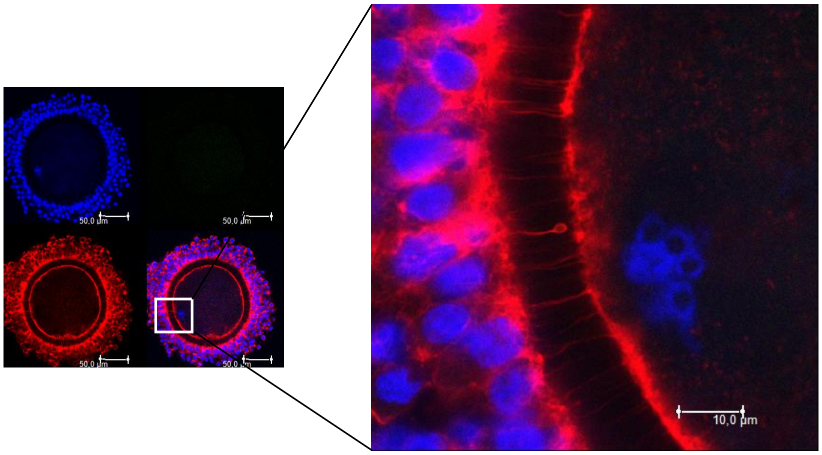

Supplementary Figure S8. Confocal photomicrographs of FABP3-negative controls without the primary antibody. No signal was detected for FABP3 immunostained using 488 Alexa Fluor (green). TZPs (actin) are stained with Alexa Fluor 647 phalloidin (red); nuclei are stained with DAPI (blue). Photomicrographs were obtained in 63 x objective. The zoomed square consists of 63 x magnification with z-stack captures with digital zoom of 3.5 x.

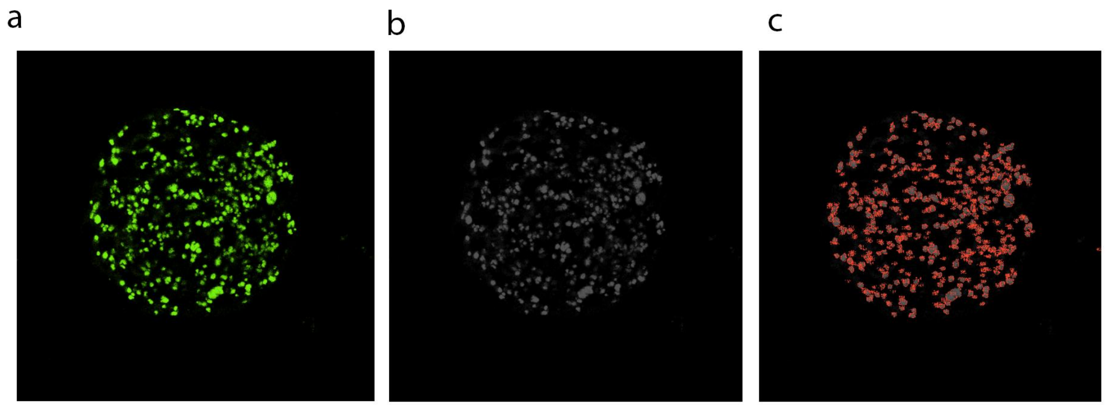

Supplementary Figure S9. Schematic images illustrating the lipid droplet quantification with BODIPY 493/503 (Molecular Probes, Eugene, OR, USA) using ImageJ plugin named “Nucleus Counter”. (a) The original image, (b) the 8 bits image and (c) image with lipid droplets individualized with “nucleus counter” plugin. “Nucleus counter” provided the area occupied by each and total lipid droplets. Then, we measured an oocyte area and we calculated a ratio “lipid droplet area/total area”.

COCs matured for 9 hours in the absence of cytochalasin B (control)

COC 1

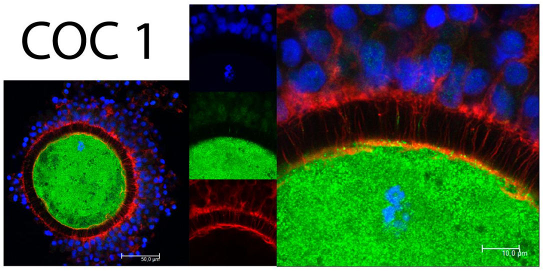

COC 2

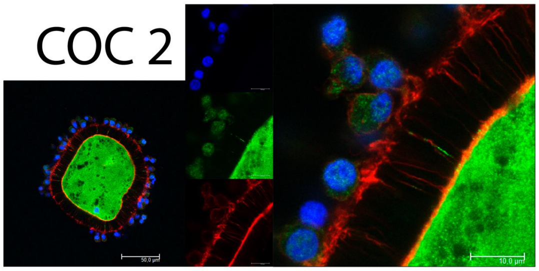

COC 3

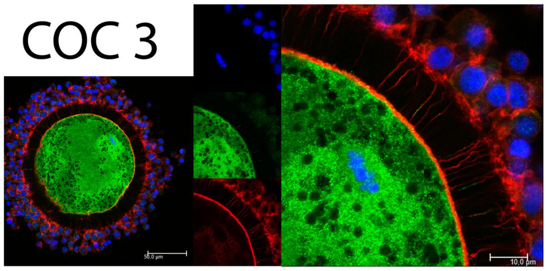

COC 4

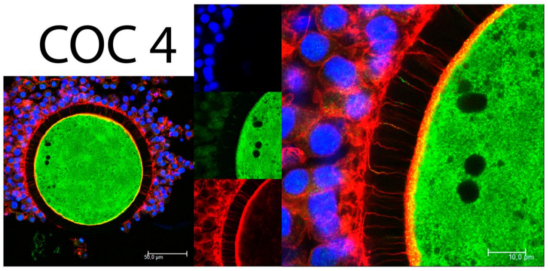

COC 5

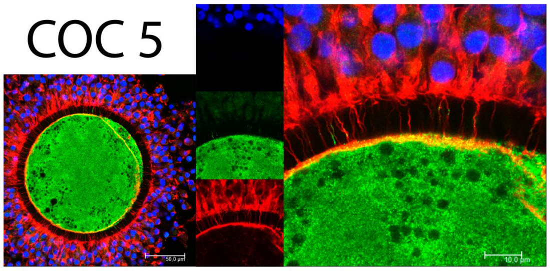

COC 6

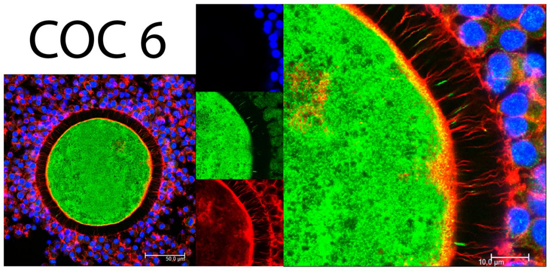

COCs matured for 9 hours in the presence of cytochalasin B

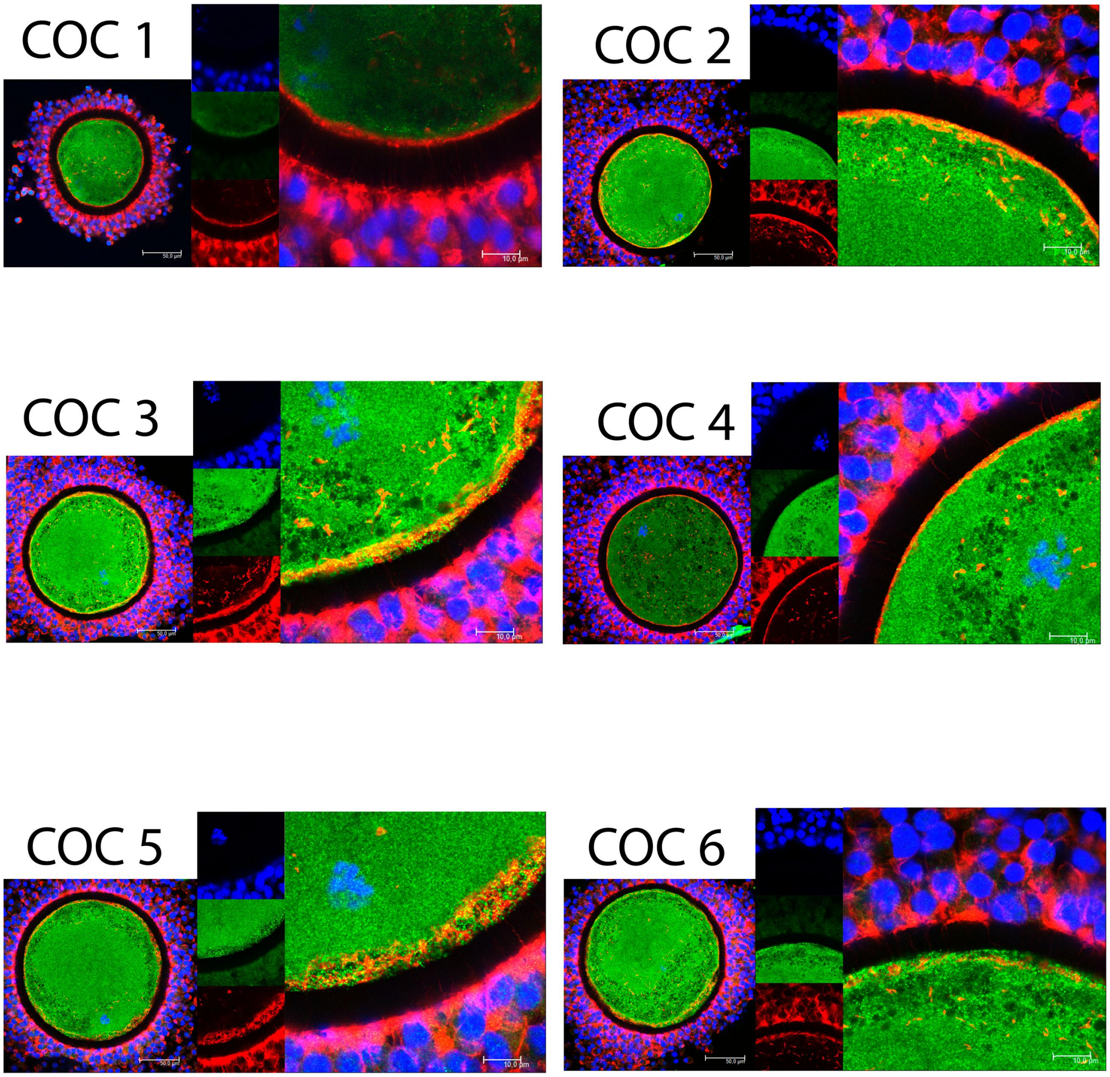

Supplementary Figure S10. Supplementary confocal photomicrographs of 6 cumulus-oocyte complexes (COCs) *in vitro*-matured for 9 hours in the absence of cytochalasin B (control) and of 6 COCs IVM for 9 hours in the presence of cytochalasin B. For each COC, the image at left was obtained in 63 x objective and is the merged z-stacked captures. The three images at the center were obtained in 63 x objective zoomed 3.5 x. From top to bottom these three images are stained to visualize: nuclei stained with DAPI (blue – the

image at the top); FABP3 immunostained using 488 Alexa Fluor (green – the image at the middle); and TZPs (actin) stained with Alexa Fluor 647 phalloidin (red – the image at the bottom). The large image at right is the digital zoom of merged z-stacked captures.

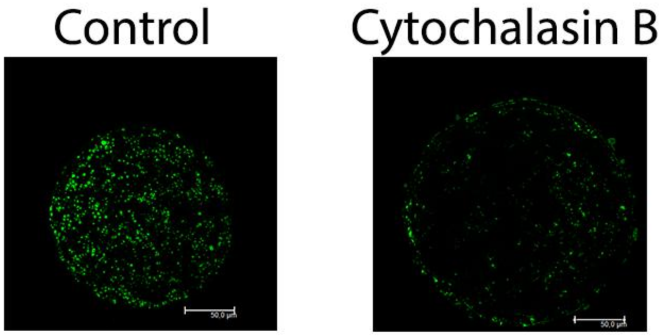

Supplementary Figure S11. Representative confocal photomicrographs of oocytes *in vitro*-matured for 9 hours in the absence (control) or presence of cytochalasin B. The oocytes were stained for lipid quantification with BODIPY 493/503 (Molecular Probes, Eugene, OR, USA). Images were captured in LSM 710 confocal microscope (Carl Zeiss) using 63 x objective.

SUPPLEMENTARY MATERIALS AND METHODS

Collection of immature, in vivo-, and in vitro-matured oocytes

A sample of immature COCs were subjected to IVM for 24 h at 38.5 °C and 5% CO_2 in air with high humidity. The IVM medium was composed of TCM-199 with Earle's salts, L-glutamine, 2.2 g/L Sodium Bicarbonate (GIBCO BRL, Grand Island, NY, USA), 0.5 μg/mL FSH (Folltropin; Ourofino Saude Animal, Cravinhos, Brazil), 50 mg/mL hCG (Vetecor; Ourofino Saude Animal), 50 μg/mL gentamicin, 0.2 mM sodium pyruvate, and 10% FBS (GIBCO). After 24 h of IVM, the oocytes were evaluated for nuclear maturation based on the presence of the first polar body (1˚PB); only cumulus cells from MII oocytes were used for further analysis.

To obtain *in vivo*-matured oocytes, we synchronized the ovarian follicular wave of cyclic Nellore cows and then subjected them to follicular superstimulation, according to the following protocol. On the first day (day 0), females received 50 mg of cloprostenol (Sincrocio, OuroFino Saude Animal, Cravinhos, Brazil), and follicular wave synchronization was induced by follicular ablation by administration of 2 mg of benzoate estradiol (Sincrodiol, Ourofino Saude Animal). Intravaginal progesterone releasing device (IPRD) implants were also placed (Sincrogest, OuroFino Saude Animal). On day 4, follicular superstimulation was initiated. A total of 133 mg of FSH (Folltropin, Bioniche Animal Health Canada INC., Chemin Georges, Lavaltrie, Canada) per cow was divided into 8 parts and administered in decreasing doses every 12 h. Along with the sixth dose of FSH, 50 mg of cloprostenol was administered and 12 h later, the IPRD was removed. On day 8, the females were administered 0.01 mg of buserelin acetate (GnRH analog,

Sincroforte, OuroFino Saude Animal). Expanded COCs were recovered after 25-26 h by ovum pick-up. *In vivo*-matured oocytes were selected based on 1˚PB extrusion and only matured oocytes (identified as MII oocytes) and the corresponding cumulus cells were used for further analysis.

All samples assigned for RNA and protein extraction were immediately placed in liquid nitrogen and stored in -80 ˚C until use.

SUPPLEMENTARY TABLE

Supplementary Table S1. Primer sequences, amplicon size and accession number of primers used for RT-qPCR reactions in oocytes and cumulus cells.

Gene Symbol	Primers	Sequence 5´-3	Amplicon size	Accession #
FABP3	FABP3 F	AAGTCAAGTCCATCGTGACGC	134	NM_174313.2
	FABP3 R	AACTGCAGTGCCATGGGTGA		
YWHAZ	YWHAZ F	GCATCCCACAGACTATTTCC	120	GU817014.1
	YWHAZ R	GCAAAGACAATGACAGACCA		
SDHA	SDHA F	AACCTGATGCTTTGTGCTCTGC	101	NM_174178.2
	SDHA R	TCGTCAACCCTCTCCTTGAAGT		
PPIA	PPIA F	CATACAGGTCCTGGCATC	108	NM_178320.2
	PPIA R	CACGTGCTTGCCATCCAA		
